# Multicentre, England-wide randomised controlled trial of the ‘Foundations’ smartphone application in improving mental health and well-being in a healthcare worker population

**DOI:** 10.1192/bjp.2022.103

**Published:** 2023-02

**Authors:** Sam N. Gnanapragasam, Rose Tinch-Taylor, Hannah R. Scott, Siobhan Hegarty, Emilia Souliou, Rupa Bhundia, Danielle Lamb, Danny Weston, Neil Greenberg, Ira Madan, Sharon Stevelink, Rosalind Raine, Ben Carter, Simon Wessely

**Affiliations:** Department of Psychological Medicine, Institute of Psychiatry Psychology and Neuroscience, King's College London, Weston Education Centre, UK and South London and Maudsley NHS Foundation Trust, UK; Department of Biostatistics and Health Informatics and King's Clinical Trials Unit, Institute of Psychiatry Psychology and Neuroscience, King's College London, UK; Department of Psychological Medicine, Institute of Psychiatry Psychology and Neuroscience, King's College London, Weston Education Centre, UK; Department of Applied Health Research, University College London, UK; Department of Occupational Health, Guy's and St Thomas’ NHS Foundation Trust, UK

**Keywords:** Randomised controlled trial, psychosocial interventions, information technologies, outcome studies, epidemiology

## Abstract

**Background:**

Healthcare workers (HCWs) have faced considerable pressures during the COVID-19 pandemic. For some, this has resulted in mental health distress and disorder. Although interventions have sought to support HCWs, few have been evaluated.

**Aims:**

We aimed to determine the effectiveness of the ‘Foundations’ application (app) on general (non-psychotic) psychiatric morbidity.

**Method:**

We conducted a multicentre randomised controlled trial of HCWs at 16 NHS trusts (trial registration number: EudraCT: 2021-001279-18). Participants were randomly assigned to the app or wait-list control group. Measures were assessed at baseline, after 4 and 8 weeks. The primary outcome was general psychiatric morbidity (using the General Health Questionnaire). Secondary outcomes included: well-being; presenteeism; anxiety; depression and insomnia. The primary analysis used mixed-effects multivariable regression, presented as adjusted mean differences (aMD).

**Results:**

Between 22 March and 3 June 2021, 1002 participants were randomised (500:502), and 894 (89.2%) followed-up. The sample was predominately women (754/894, 84.3%), with a mean age of 44⋅3 years (interquartile range (IQR) 34–53). Participants randomised to the app had a reduction in psychiatric morbidity symptoms (aMD = −1.39, 95% CI −2.05 to −0.74), improvement in well-being (aMD = 0⋅54, 95% CI 0⋅20 to 0⋅89) and reduction in insomnia (adjusted odds ratio (aOR) = 0⋅36, 95% CI 0⋅21 to 0⋅60). No other significant findings were found, or adverse events reported.

**Conclusions:**

The app had an effect in reducing psychiatric morbidity symptoms in a sample of HCWs. Given it is scalable with no adverse effects, the app may be used as part of an organisation's tiered staff support package. Further evidence is needed on long-term effectiveness and cost-effectiveness.

## Background

Healthcare workers (HCWs) have faced considerable pressures during the COVID-19 pandemic. This includes work-based ones such as the risk of infection, increased workload, exposure to trauma, social challenges of isolation, increased caring demands and loss of loved ones. While there has been a substantial psychological toll across the entire population, some (but not all) studies suggest that HCWs have elevated levels of probable common mental disorders, anxiety, depression and insomnia.^1–4^ Poor mental health status has ramifications for not only HCWs and their families, but also HCWs’ ability to support patients.

Health systems and organisations have tried to support staff during the pandemic through a range of initiatives, however, most lack evidence.^5^ Smartphone applications (apps) are one type of support offered. Evidence available pre-pandemic across different population and occupational groups suggest that smartphone apps can help improve symptoms of general psychiatric morbidity, depression, anxiety and well-being with small effects (Hedge's *g* 0.24–0.33),^6–10^ and with moderate effects for insomnia (Hedge's *g* = 0.70).^6^ The studies notably highlight caution; namely, the need for robust adequately powered trials, and interventions evaluated in the context of a target population, such as HCWs, where evidence is scarce. The lack of evidence in the HCW population is important, as non-evidence-based interventions may not only be unhelpful but also have the potential to offer false hope, cause harm and lack value-for-money. This is particularly important given the vast numbers of unvalidated mental health apps on offer in the wider market.^11^

Our primary aim was to determine the effectiveness of the Foundations app on general (non-psychotic) psychiatric morbidity (herein psychiatric morbidity). Secondary aims were to assess the effect of this app on anxiety symptoms, depressive symptoms, functioning, sleep, well-being, resilience and presenteeism.

## Method

### Study design and participants

We conducted this England-wide multicentre parallel-group randomised (1:1) controlled trial of both acute hospital providers and mental health providers to compare the app to a wait-list control group over an 8-week follow-up period. The study was nested in a prospective cohort study that studied the health and well-being of HCWs across 18 NHS England trusts during the COVID-19 pandemic, between March 2020 to January 2021. Further details are outlined in the study protocol article published elsewhere.^12^

### Ethics

The study was registered (EudraCT Number: 2021-001279-18) and the protocol published on the NHS CHECK study website (Supplementary Material 1 available at https://doi.org/10.1192/bjp.2022.103). The authors assert that all procedures contributing to this work comply with the ethical standards of the relevant national and institutional committees on human experimentation and with the Helsinki Declaration of 1975, as revised in 2008. All procedures involving human participants were approved by Health Research Authority Research Ethics Committee (study ethical approval number 20/HRA/2107, IRAS: 282686).

### Eligibility

The inclusion criteria: participants were NHS-affiliated members of staff; able to communicate in English; had access to the internet; owners of a smartphone with access to the Apple or Google app stores; were 18 years of age or older. The exclusion criteria were: if they reported to have any plans to start any new interventions during the 8-week trial period (i.e. any well-being apps, psychological therapies and pharmacological therapies).

### Participant selection

HCWs from 16 NHS trusts who had previously agreed to be contacted for further research in the NHS CHECK prospective cohort study were invited at random.^12^ The email included a link to the participant information sheet, consent form and baseline assessment, with one follow-up email sent for those that did not respond to the initial invitation. Following participant consent the baseline assessments were completed online prior to randomisation by an unmasked member of the research team.

### Randomisation sequence generation and allocation procedure

The randomisation sequence was generated using a varying permuted block design in a 1:1 ratio stratified by NHS trust (site) and occupational role (clinical or non-clinical). The sequence was concealed on a user-controlled web-based King's Clinical Trials Unit (KCTU) system. The sequence was allocated by members of the research team following baseline data completion.

### Masking

Trial participants were unmasked. The senior statistician (B.C.), co-investigators (N.G., S.S., I.M., D.L.) and one chief investigator (S.W.) were masked. The trial statistician (R.T.-T.) was masked until the statistical analysis plan was approved, then was pseudo-masked (able to see outcome data coded A/B) following KCTU standard operating procedures (SOPs). Participant data was stored in separate databases from the randomisation data and held by four fully unmasked researchers (H.R.S., S.H., E.S. and R.B.).

### Intervention

Participants allocated to the control group received usual care. After the end of the trial, participants in the control group were given access to the app no earlier than 7 days after the final assessment time point. Participants allocated to the app were provided access by an unmasked researcher via email inviting them to download the app.

The app seeks to promote behaviour change and positive well-being habits, and is designed to promote mental well-being, manage stress and improve sleep. Active use (ideally daily) is encouraged during weeks 1 and 2 to gain and develop such well-being habits and skills. Users are prompted and encouraged to continue with these behaviours via smartphone home screen notifications from the app.

The user selects one of six focus areas to work on during onboarding (relaxation, sleep, anxious thoughts, feeling down, self-esteem, stress). The choice of focus area influences the programmes and activities that are recommended to the user on the homepage widgets of the app. The user can change their focus area at any time. Content can be selected from either the homepage recommendations or from the content library. In the library, content is organised by focus area (see above in addition to ‘hot topics’, ‘relating to others’ and ‘healthy lifestyle’) and by technique.

The techniques included are: relaxation (for example breathing exercises such as 4–7–8 breathing, diaphragmatic breathing, progressive muscle relaxation), working with thoughts (for example cognitive–behavioural therapy (CBT)-based cognitive restructuring, postponing worry), positive thinking (gratitude journaling on people, achievements, gratitude), mindfulness and mediation, sleep relaxations (guided audios, ambient sounds, soundscapes), working on sleep (CBT for insomnia including sleep hygiene and scheduling), physical health (healthy habits, desk exercises and physical activity programmes), or tips from the experts.

The app includes a range of interactive standalone activities and programmes. See Supplementary Tables 1 and 2 for app content and Supplementary Fig. 1 for the app interface. A programme is a sequence of activities aiming to develop a skill or technique. Activities are delivered through different formats including journaling and reflection, games/quizzes, audios and learning articles. As an example, the ‘working with thoughts’ programme starts with psychoeducation on automatic thoughts and the principles of CBT. The next activity encourages the user to journal their thoughts and feelings and keep a thought record in the app. In the next activity, the user is provided with psychoeducation and a quiz on common cognitive distortions. In the final activities, the user can review their thought record and create balanced thoughts.

The app was commercially developed by Koa Health prior to the study design, and evaluated in this independent randomised controlled trail (RCT). The app was developed from evidence-based techniques including CBT, mindfulness-based CBT, relaxation techniques and positive psychology. It was created through user-centred design and co-creation techniques. Content areas were derived from large-scale surveys and features scoped through qualitative concept and usability testing and feedback. The app was developed around the COM-B framework to give users the capability, opportunity and encourage motivation for sustained behaviour change.^13^ Capability is the component the application primarily focuses on – providing psychoeducation on mental well-being, while also supporting users through programmes to build positive coping skills and proactive well-being strategies. The accessibility of digital interventions gives the opportunity for users to work autonomously on a range of techniques dependent on their needs. Motivation is supported through user-centred design principles that enhance engagement to interact, use and apply the appropriate health behaviours suggested in the app.

### Outcomes

The primary outcome was the 12-item General Health Questionnaire (GHQ-12), which detects symptoms of general (non-psychotic) psychiatric morbidity^14^ including social dysfunction, anxiety and loss of confidence.^15^ Using Likert scoring, scores range from 0 to 36 with higher scores indicative of worse mental health. This measure has well-validated psychometric properties, with high internal consistency (Cronbach's alpha 0.90), and good discrimination validity (delta  0.94).^15^

Secondary continuous outcomes included: the Brief Resilience Scale (BRS), a 6-item participant self-report measure, to assess an individual's ability to bounce back or recover from stress.^16^ The converted 7-item Short Warwick-Edinburgh Mental Well-being Scale (SWEMWBS) assesses subjective well-being and psychological functioning compared with the general population (all items are worded positively and address aspects of positive mental health).^17^ The 6-item Stanford Presenteeism Scale (SPS-6) continuous total score assesses subjective impact of a worker's perceived ability to concentrate on work tasks despite health impairments.^18^

Secondary binary outcomes included: the 7-item Generalized Anxiety Disorder (GAD-7)^19^ and the 9-item Patient Health Questionnaire (PHQ-9),^20^ both with a cut-off of ≥10 to indicate moderate-to-severe anxiety or depression;^5^ the 5-item Work and Social Adjustment Scale (WSAS),^21^ using a score of ≥21 to suggest moderately severe psychopathology; the 3-item Minimal Insomnia Symptom Scale (MISS),^22^ with a binary cut-off of 6 indicating severe insomnia.

Psychological (including other apps) support, pharmacological support and COVID-19 stressors were assessed. Stressors considered included loss of family income; problems managing finances; own illness because of COVID; illness of family member or friend because of COVID; bereavement because of COVID; caring role for child/children; caring role for dependent.

Assessments took place at baseline (prior to downloading the app), then week 4 and week 8. Participants received the assessments online with up to two email reminders. Participants in both arms were given a £25 gift voucher for each assessment completed. Participants were asked to contact the study team to report any adverse events experienced. Adverse events were defined in this study as negative unintended consequences of using the app (including not seeking other care or support) that led to injury, impairment or other harm.^23^

### Sample size justification

Prior research of apps indicated a small effect size of 0.3,^6–10^ so with a type 1 error = 0⋅05, to detect this difference with 80% power we would need 352 participants followed-up. We originally planned to randomise 700 participants to account for an attrition rate of approximately 50%.^24^ Early during the trial, (prior to any outcome assessment) we found a 60% fidelity rate (owing to participants not downloading the app), so we inflated the sample size to randomise 1000 participants to account for this.

### Statistical analysis

The statistical analysis plan was first approved on 11 May 2021 and all updated versions uploaded; the final version 1.4 was approved on 3 August 2021 (Supplementary Material 2). All statistical analysis plan versions were drafted by a masked senior statistician (B.C.). Week 4 and week 8 assessments were cleaned by a masked trial statistician (R.T.-T.), who became partially masked on 27 July 2021. The final follow-up was on 2 August 2022. The study followed KCTU SOPs.

The primary analysis used a multilevel multivariable linear mixed-effect model at both follow-up time points. Mixed-effects models were used to account for the repeated time points.^25^ Fixed effects were fitted for time point, treatment, the baseline measure of the outcome, age, gender, ethnicity, occupational role and use of another mental health intervention (smartphone app, medication or psychological talking therapy) and a random intercept for participant and site and was fitted using restricted maximum likelihood estimation. Between-group adjusted mean differences (aMD) for the difference between the app and control were estimated with associated 95% CI presented alongside *P*-values.

Secondary continuous outcomes were analysed identically. Secondary binary outcomes that used moderate-to-severe symptom thresholds were analysed with a multilevel logistic regression in the same framework. The senior statistician (B.C.) remained masked until all checks had been carried out on the validity of the modelling assumptions, following KCTU SOPs. Standardised effects are presented in the figures to compare outcomes. Stata version 16 was used throughout.^26^

### Analysis population and sensitivity analyses

A modified intention-to-treat (mITT) population was used. This included all randomised participants with at least one follow-up assessment irrespective of whether they downloaded the app.

We carried out two planned sensitivity analyses evaluating the impact of drop out from those randomised and the mITT using pattern mixture models, and inverse probability weighting, as well as a re-analysis of the primary outcome using the following definition of the per-protocol-population (excluding participants with inadequate app usage).

We carried out an additional *post hoc* sensitivity analysis to assess the between-group difference after additionally adjusting for insomnia in the primary analysis.

### Subgroup analyses

The primary outcome was assessed within each demographic and clinical characteristic subgroup presenting the aMD. We investigated the effect observed under adherence to the protocol under four conditions, participants who: downloaded the app; completed two activities per week; completed four activities per week; completed one programme per week.

## Results

**Fig. 1:**
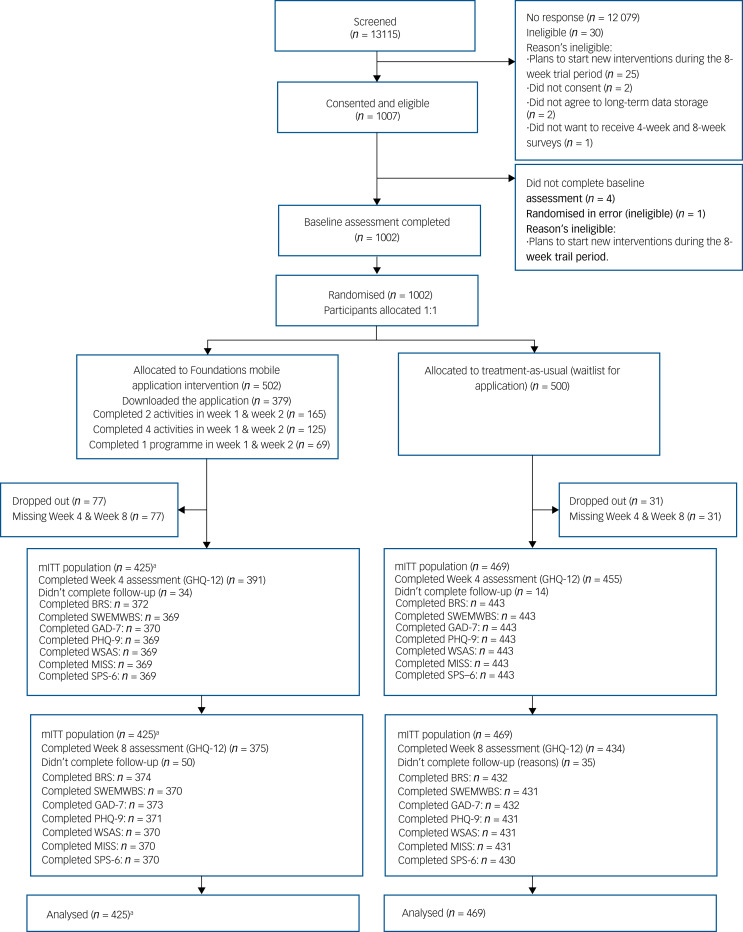
CONSORT diagram. GHQ-12, 12-item General Health Questionnaire; BRS, Brief Resilience Scale; SWEMWBS, Short Warwick-Edinburg Mental Well-being Scale; GAD, 7-item Generalized Anxiety Disorder; PHQ-9, 9-item Patient Health Questionnaire; WSAS, Work and Social Adjustment Scale; MISS, Minimal Insomnia Symptom Scale; SPS-6, Stanford Presenteeism Scale. a. One participant missing baseline ethnicity not included in adjusted models.

Participants were enrolled from 16 NHS trusts across England between 18 March and 2 June 2021. In total, 1002 participants were randomised, 502 in the app group and 500 in the control group (CONSORT, (Fig. 1). Of these, 894 featured in the mITT population since 108 participants (10⋅8%) (*n* = 77 in the app group and *n* = 31 in the control group) did not report any post-baseline assessment data (Supplementary Table 3). There was minimal difference in the characteristics of the participants not included in the mITT population (Supplementary Tables 3–5). The findings are reported consistent with the CONSORT statement.

We followed-up 846 and 809 participants at week 4 and 8. The sample was predominately women (754/894, 84.3%), and with a mean age of 44.3 years (range 20–76 (interquartiIe range (IQR) 19)), most were White (*n* = 817/894, 91.39%) and worked in a clinical role 61% (544/894), (Table 1). The two randomised groups were balanced at baseline (Table 1).

**Table 1:** Baseline demographics and characteristics of the intention-to-treat population

	App group (*n* = 425)	Control group (*n* = 469)	Total (*n* = 894)
Age, years			
18–29	65 (15.29)	55 (11.73)	120 (13.42)
30–39	85 (20.00)	104 (22.17)	189 (21.14)
40–49	117 (27.53)	143 (30.49)	260 (29.08)
50–59	114 (26.82)	123 (26.23)	237 (26.51)
≥60	44 (10.35)	44 (9.38)	88 (9.84)
Gender			
Women	356 (83.76)	398 (84.86)	754 (84.34)
Men	67 (15.76)	68 (14.50)	135 (15.10)
Prefer not to say	2 (0.47)	3 (0.64)	5 (0.56)
Ethnicity			
White	389 (91.53)	428 (91.26)	817 (91.39)
Black/African/Caribbean/Black British	7 (1.65)	6 (1.28)	13 (1.45)
Asian/Asian British	15 (3.53)	24 (5.12)	39 (4.36)
Mixed/multiple and other racial and ethnic groups	13 (3.06)	11 (2.34)	24 (2.68)
Missing	1 (0.24)	0 (0.0)	1 (0.11)
Clinical occupational role	251 (59.06)	293 (62.47)	544 (60.85)
Dietitian	0 (0)	2 (0.68)	2 (0.37)
Doctor	25 (9.96)	23 (7.85)	48 (8.82)
Healthcare assistant/nursing assistant	36 (14.34)	44 (15.02)	80 (14.71)
Healthcare Scientist (lab technician)	1 (0.4)	3 (1.02)	4 (0.74)
Medical associate/assistant	2 (0.8)	1 (0.34)	3 (0.55)
Midwife	3 (1.2)	6 (2.05)	9 (1.65)
Nurse	95 (37.85)	115 (39.25)	210 (38.6)
Occupational therapist	11 (4.38)	12 (4.1)	23 (4.23)
Other	39 (15.54)	30 (10.24)	69 (12.68)
Pharmacist/pharmacy technician	4 (1.59)	6 (2.05)	10 (1.84)
Physiotherapist	6 (2.39)	11 (3.75)	17 (3.13)
Psychologist/assistant psychologist	23 (9.16)	28 (9.56)	51 (9.38)
Radiographer	3 (1.2)	5 (1.71)	8 (1.47)
Speech and language therapist	1 (0.4)	4 (1.37)	5 (0.92)
Ward manager	2 (0.8)	3 (1.02)	5 (0.92)
Non-clinical occupational role	174 (40.94)	176 (37.53)	350 (39.15)
Administrative and clerical	87 (50.00)	88 (50.00)	175 (50.00)
Catering services	2 (1.15)	0 (0.00)	2 (0.57)
Chaplaincy	1 (0.57)	1 (0.57)	2 (0.57)
Clinical support	4 (2.30)	6 (3.41)	10 (2.86)
Domestic services	2 (1.15)	3 (1.70)	5 (1.43)
Estates services	0 (0.00)	3 (1.70)	3 (0.86)
Finance	1 (0.57)	4 (2.27)	5 (1.43)
Healthcare scientists	6 (3.45)	10 (5.68)	16 (4.57)
Human resources	2 (1.15)	6 (3.41)	8 (2.29)
IT support	5 (2.87)	6 (3.41)	11 (3.14)
Management	26 (14.94)	20 (11.36)	46 (13.14)
Other	21 (12.07)	15 (8.52)	36 (10.29)
Research/academic	12 (6.90)	11 (6.25)	23 (6.57)
Social services (social worker)	2 (1.15)	0 (0.00)	2 (0.57)
Support services (driver/porter/secretary)	3 (1.72)	3 (1.70)	6 (1.71)
Use mental health/well-being app at least once a week			
No	372 (87.53)	412 (87.85)	784 (87.70)
Yes	53 (12.47)	57 (12.15)	110 (12.30)
Use of mental health medication			
No	308 (72.47)	359 (76.55)	667 (74.61)
Yes	117 (27.53)	110 (23.45)	227 (25.39)
Receiving therapy			
No	400 (94.12)	433 (92.32)	833 (93.18)
Yes	25 (5.88)	36 (7.68)	61 (6.82)
Country of birth			
UK	364 (85.65)	414 (88.27)	778 (87.02)
European Union	26 (6.12)	17 (3.62)	43 (4.81)
Other	33 (7.76)	37 (7.89)	70 (7.83)
Missing	2 (0.47)	1 (0.21)	3 (0.34)

### Primary outcome

**Fig. 2:**
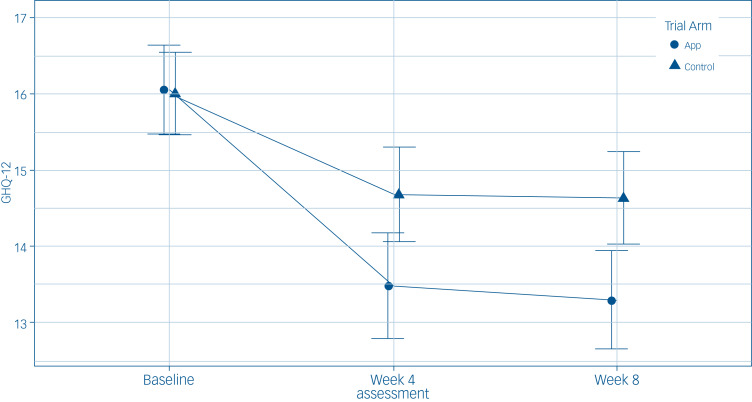
Temporal effects of the general psychiatric morbidity (12-item General Health Questionnaire (GHQ-12)) outcome assessment treatment group mean, per time point with associated 95% confidence intervals for the intention-to-treat population (*n* = 894). App, app group; Control, control group.

The reduction of symptoms was similar at 4 weeks and 8 weeks in the app group compared with the control group (Fig. 2). The app was associated with a significant between-group reduction in psychiatric morbidity symptoms in the primary analysis (aMD = −1.39, 95% CI −2.05 to −0.74). There was no evidence of a time × app group interaction (*P* = 0.49).

### Secondary outcomes

**Fig. 3:**
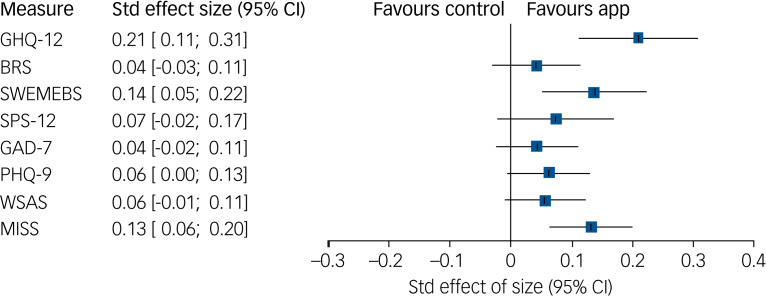
Standardised effect sizes with associated 95% confidence intervals for between-group comparison up to week 8 (accounting for both follow-up time points) from the adjusted analyses. Std, standard; GHQ-12, 12-item General Health Questionnaire; BRS, Brief Resilience Scale; SWEMWBS, Short Warwick-Edinburg Mental Well-being Scale; SPS-6, Stanford Presenteeism Scale; GAD, 7-item Generalized Anxiety Disorder; PHQ-9, 9-item Patient Health Questionnaire; WSAS, Work and Social Adjustment Scale; MISS, Minimal Insomnia Symptom Scale.

The app was associated with an increase in the SWEMWBS (aMD = 0.54; 95% CI 0⋅20–0⋅89); Table 2). There was a 64% reduction in the odds of insomnia (MISS) in the app group (adjusted odds ratio (aOR) = 0⋅36, 95% CI 0⋅21 to 0⋅60). There was no association between the app group and: BRS (aMD = 0⋅03, 95% CI −0⋅03 to 0⋅09); presenteeism (SPS-6, aMD = 0⋅38, 95% CI −0⋅12 to 0⋅87); moderate anxiety (GAD-7, aOR = 0⋅69, 95% CI 0⋅39 to 01.23); moderate depression (PHQ-9, aOR = 0⋅61, 95% CI 0⋅35 to 1.04); moderately severe or severe functioning impairment (WSAS, aOR = 0⋅61, 95% CI 0⋅33 to 1.11). No adverse events were reported to the trial team. Standardised effect sizes can be seen in Fig. 3.

**Table 2:** Crude and adjusted analysis multilevel, multivariable linear regression (and logistic regression) of the between-group comparison during follow-up (accounting for both time points)^a^

Outcome and measure	*n*	OR (95% CI)	MD (95% CI)	*P*	aOR^b,c^ (95% CI)	aMD^b,c^ (95% CI)	*P*
Primary outcome							
GHQ-12	**894**	**–**	**−1.34 (−2.15 to −0.53)**	**0.001**		**−1.39 (−2.05 to −0.74)**	**<0.001**
Secondary outcomes (continuous)							
BRS	871	–	0.06 (−0.05 to 0.16)	0.29	–	0.03 (−0.03 to 0.09)	0.26
SWEMWBS	**867**	–	0.45 (−0.06 to 0.95)	0.08	**–**	**0.54 (0.20 to 0.89)**	**0.002**
SPS-6	865	–	0.45 (−0.18 to 1.08)	0.16	–	0.38 (−0.12 to 0.87)	0.14
Secondary outcomes (binary)							
GAD-7	870	0.70 (0.36 to 1.35)	–	0.29	0.69 (0.39 to 1.23)	–	0.21
PHQ-9	867	0.70 (0.39 to 1.28)	–	0.25	0.61 (0.35 to 1.04)	–	0.07
WSAS	866	0.76 (0.39 to 1.50)	–	0.43	0.61 (0.33 to 1.11)	–	0.10
MISS	**865**	**0.35 (0.20 to 0.64)**	**–**	**0.001**	**0.36 (0.21 to 0.60)**	**–**	**<0.001**

a, adjusted; GHQ-12, 12-item General Health Questionnaire; BRS, Brief Resilience Scale; SWEMWBS, Short Warwick-Edinburg Mental Well-being Scale; SPS-6, Stanford Presenteeism Scale; GAD, 7-item Generalized Anxiety Disorder; MD, mean difference; PHQ-9, 9-item Patient Health Questionnaire; WSAS, Work and Social Adjustment Scale; MISS, Minimal Insomnia Symptom Scale.

a. Presenting the MD and aMD from the linear regression for: general (non-psychotic) psychiatric morbidity (GHQ-12); resilience (BRS); well-being (SWEMWBS); and presenteeism (SPS). As well as the odds ratio and aOR of: moderate anxiety symptoms (GAD-7 ≥ 10); moderate depression symptoms (PHQ-9 ≥ 10); moderately severe or severe functioning impairment (WSAS ≥ 21); and insomnia (MISS ≥ 6) from the logistic regression models.

b. Adjusted for: baseline outcome, age, gender, ethnicity, occupational role (clinical or non-clinical), use of other mental health application (yes or no), use of mental health/well-being medication (yes or no), use of psychological or talking therapy (yes or no), and 4-week and 8-week assessments.

c. One participant not included in the analysis because of data missing on ethnicity.

From the subgroup analyses the following were seen to have larger effects for the primary outcome (Supplementary Fig. 2): women, clinical staff and younger participants. However, we note no statistically significant effect in men, non-clinical staff and those aged ≥40 years. Caution should be taken when interpreting underpowered subgroup analysis.

Of the 108 participants that were excluded from the mITT population a significant association of drop out was found only for participants who were current users of mental health medication (Supplementary Table 3). The use of medication was fitted using an inverse probability weighting and there was no change in the overall study findings (aMD = −1⋅40, 95% CI −2⋅05 to −0⋅75). Overall, 379 of the 502 app participants (75.5%) downloaded the app with a median of 46.8 min spent using the app over the 8 weeks (interquartile range (IQR) 16.6−129.8, Supplementary Tables 6 and 7). The per-protocol-population was defined using app usage four ways and all offered consistent effects with the primary analysis (Supplementary Tables 8).

We carried out an additional *post-hoc* sensitivity to assess the association after additionally accounting for the effect of insomnia in the primary analysis, and the findings were unchanged for the primary outcome (aMD = −0⋅85, 95% CI −1⋅43 to −0⋅28).

## Discussion

### Main findings

The study found that the use of the app by a sample of HCWs in England was associated with an improvement in mental health and well-being. We found a statistically significant reduction in symptoms of general psychiatric morbidity and insomnia as well as an increase in mental well-being. On the other hand, we did not note any statistically significant improvement in symptoms of depression, anxiety, resilience, presenteeism and functioning.

### Comparison with findings from other studies

The improvement found in general psychiatric morbidity is in-keeping with the small positive effect seen with cognitive behavioural and mindfulness-based apps used in clinically symptomatic, undifferentiated general population and non-health worker occupational groups.^8,27^ This is notable given the scarce and mixed evidence to date for HCWs, with recent RCTs of digital interventions during the pandemic having had mixed results and relying on 2 weeks of follow-up at most.^28,29^ Although the effect size for improvement in general psychiatric morbidity is modest, given the study population was not targeted and spanned the clinical severity spectrum, including those with no symptomology, the potential effect at a population level as part of a universal offer is considerable.^30^ The ability to shift population symptom distributions away from the diagnostic threshold is promising given apps such as this one can be scaled at pace, offered widely and provide easy-to-access and time-flexible support.

The reduction in symptoms of insomnia is consistent with evidence from occupational contexts that show moderate effect.^6^ Strong effects, not found in this study, are reported in app studies undertaken in clinically symptomatic groups with insomnia.^31,32^ The small effect and improvement in well-being is in-keeping with findings from previous occupational digital mental health interventions.^6,7^ However, our study did not find a significant overall effect for depressive and anxiety symptoms, unlike some digital interventions that had a small effect.^6–10^ Given this trial's broad inclusion criteria, which included participants with and without moderate–severe threshold symptom severity, our trial notably varied from comparative studies that only included participants with clinical disorders. Our trial evaluated a universal rather than a targeted offer. Further, given that a binary classification for depression and anxiety symptoms was used in analysis (see Method section related to PHQ-9 and GAD-7), our findings may not have identified subthreshold improvements if they were present.

### Interpretation of our findings

Although there is some overlap between measures (e.g. GHQ-12 and PHQ-9 have affective questions), we found the app to be associated with statistically significant improvement in some, but not all. This may be because of the type and nature of questions. For example, GHQ-12 considers respondent's current state across several domains and how it differs from usual state, whereas PHQ-9 asks for depression symptom frequency in the preceding 2 weeks. In addition to the primary outcome showing a benefit, the effect estimates for depression, anxiety, presenteeism, resilience and impairment of functioning all favoured the app, albeit with a reduced and non-significant effect. These findings may be owing to underpowered analyses.

We found that being women, younger and a clinical staff member were associated with greater reductions in the primary outcome of general psychiatric morbidity. This association was also true for those who reported exposure to a potentially morally injurious event. These findings are of relevance as prevalence studies during the pandemic suggest that younger staff, women, clinical HCWs such as nurses, and those who report exposure to potentially morally injurious events have higher rates of probable common mental disorders.^2,3^ However, we note no statistically significant effect in men, non-clinical staff, and those aged ≥40 years. This underlines the need for further powered study of subgroups and to ensure that where offered, validated smartphone apps are one among a set of validated support interventions provided to HCWs. We used several behavioural change techniques that were readily applicable via an app. It may be that other barriers to the specified behaviours exist, that could be better targeted through other techniques. Future work to further improve the app might usefully explore additional components to include, and identify them using the COM-B and theoretical development framework models.^13,33^

The app was used across 8 weeks with a median use of 46.8 min. App use was highest in week 1 and decreased week on week during the trial period (Supplementary Tables 7 and 8). This is in-line with a systematic review that found approximately 70% of users stopped using apps after 6 weeks.^34^ Our study found a consistent effect at week 4 and week 8 despite decline in use. This is congruous with the app design as described in the Method section whereby individuals are expected to learn skills and develop positive well-being habits that can be applied without ongoing app use. However, given the small effect size and gradual decline in app use, outstanding questions remain regarding whether the results may at least in part be accounted for by digital placebo effects.^35^ In addition, outstanding questions remain regarding whether the beneficial effects noted are maintained beyond the 8-week study period, and if so, for how long.

### Strengths and weaknesses

Our RCT has a number of strengths. First, to our knowledge this is the largest study of a mental health and well-being app in a HCW population, and is one of the few studies to evaluate interventions (digital or otherwise) supporting HCWs.^5^ Second, the sample offered an external generalisable HCW population who could be offered and use such an intervention. The sample included both clinical and non-clinical staff working at acute hospital and mental health providers. This is notable as studies of apps are often restricted to a specific clinical sample (targeted rather than universal). Third, prevalence of mental ill health found at baseline was consistent with other studies of HCWs in England.^2,3^ Finally, this study had a very high follow-up rate (84.4% in week 4 and 80.7% in week 8).

Our study has several limitations. First, the sample was predominantly women and White (84% and 91%, respectively) when compared with NHS demographics (77% and 76%, respectively).^36^ Second, recruiting participants from the NHS CHECK cohort study as a nested trial meant that we were recruiting from a cohort of people already taking part in research. The low conversation rate between those screened and randomised may limit the generalisability of the sample. Reassuringly however, the NHS CHECK cohort is the only known study with a representative sample of HCWs having used a robust sample frame based on organisational human resources data rather than word of mouth invitations/convenience sampling.^12^ Third, given this was a waiting-list controlled trial, the group effects seen in the intervention arm as compared with treatment as usual wait-list control arm may have been inflated.^37^ Fourth, the mental health of the participants was evaluated through self-reported online instruments, which favour sensitivity over specificity and may overestimate prevalence, rather than gold standard diagnostic clinical interviews.^38^ Finally, although this study has a long follow-up relative to other app studies of HCWs and other occupational groups, the 8-week follow-up limits the ability to understand the longer-term impact of app use.

In conclusion, our study suggests that the app was of modest benefit with no adverse effects for a sample of HCWs in England. Although the effect of the app on general psychiatric morbidity was small, its potential reach across a whole population of healthcare staff is considerable. This is promising when we consider that apps have the potential to mitigate barriers typically faced by HCWs in accessing traditional forms of support such as lack of anonymity and shift-work related time constraints. Given the modest effect size and variations across different demographic groups, there is need for caution in use. When offered to employees, the app should be part of an organisation's tiered staff support package to cater for different pathways to care, utilisation, preferences and disease severities. Future work is needed to examine longer-term effectiveness. There is also a need to determine how the app improves outcomes, evaluate the cost-effectiveness and identify the characterisations of which HCWs use, and do not use, such apps.

## Data Availability

Data will be available to other researchers who provide a justified hypothesis and structured statistical analysis plan addressing a legitimate research question that is approved by the Trial Management Group and data sharing agreement approved. Only deidentified participant data will be provided.

## Supplementary material

For supplementary material accompanying this paper visit https://doi.org/10.1017/bjp.2022.103. click here to view supplementary material
