# Supplementary material for: Multicentre, England-wide randomised controlled trial of the ‘Foundations’ smartphone application in improving mental health and well-being in a healthcare worker population
Source: Br J Psychiatry. 2023 Feb;222(2):58–66. doi: 10.1192/bjp.2022.103 (PMC10895508; doi:10.1192/bjp.2022.103)
Supplement: Supplementary file 1 [file S0007125022001039sup.zip › S0007125022001039sup004.docx]

Supplementary Material

### Figure 1: Application interface


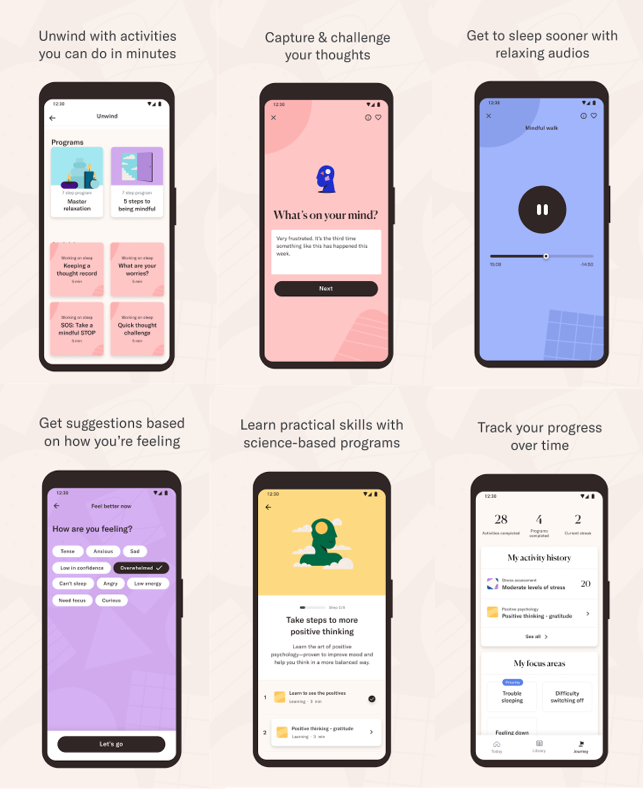


### Figure 2: Subgroup Analysis plot for the primary outcome


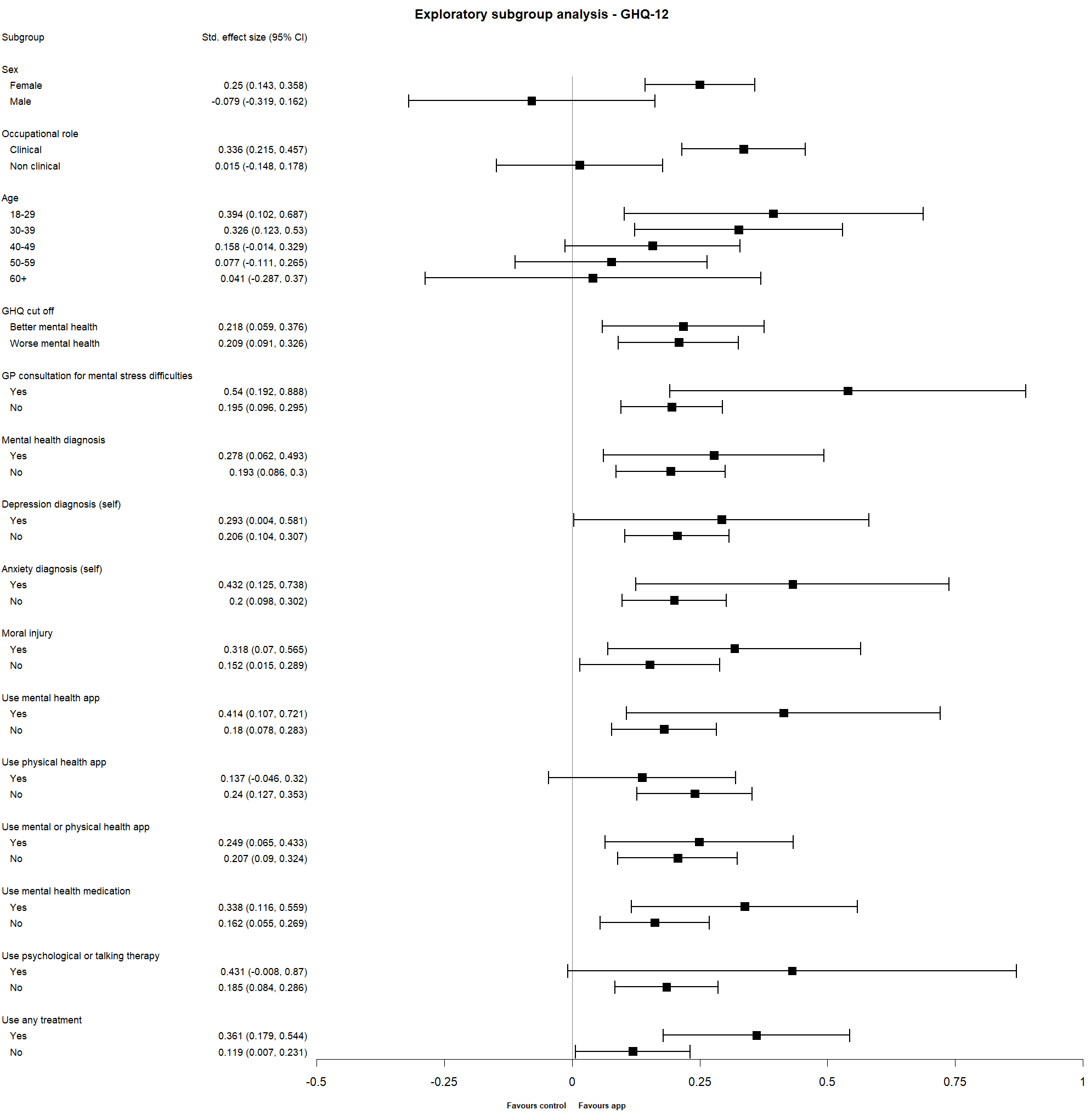


### Figure 3: Temporal effects of outcome assessments treatment group mean per timepoint with associated 95% confidence intervals for the intention to treat population (BRS, SWEMWBS and SPS-6)


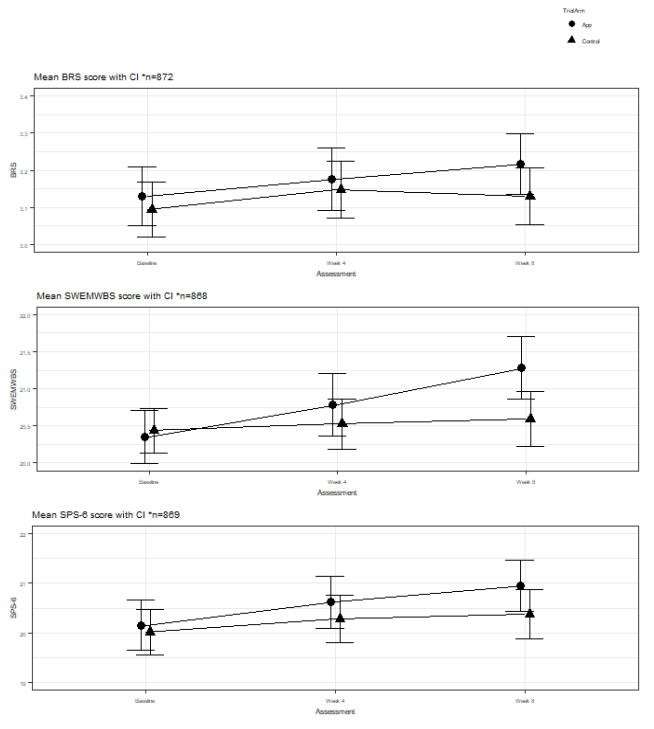


### Figure 4: Temporal effects of outcome assessments treatment group mean per timepoint with associated 95% confidence intervals for the intention to treat population (PHQ-9, GAD-7, WSAS and MISS)


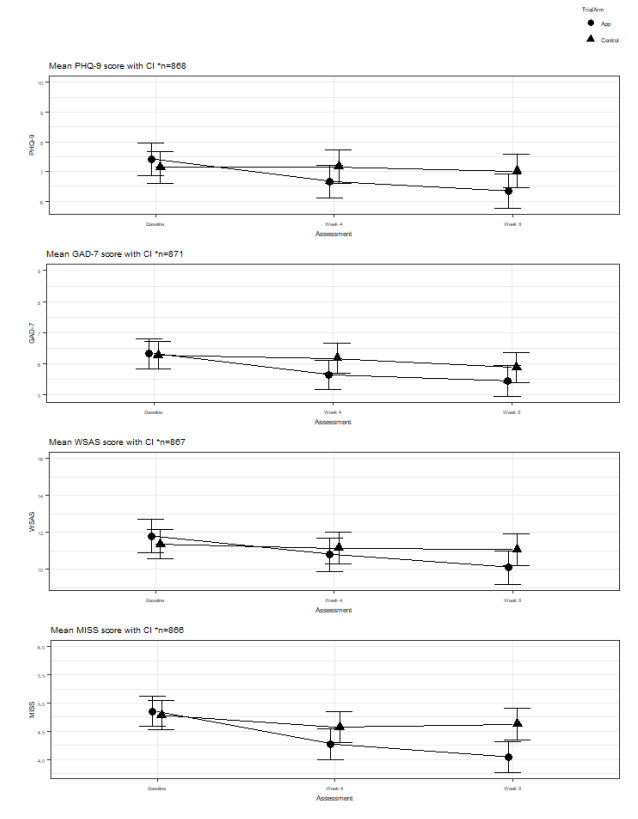


### Table 1: App content number and format

| Programmes AND Activities | Number |
| --- | --- |
| Total Programmes | 19 |
| Total Activities in Programmes | 108 |
| Standalone Activities (not in a programme) | 85 |
| All Activities (both in and out of programmes) | 193 |
| Format of ACtivities |  |
| Article: Reading | 33 |
| Slides: Reading | 50 |
| Add/Label Record: Journaling | 21 |
| Question Record: Reflection on Journal Entries | 9 |
| Record Review: Record of Journal Entries | 4 |
| Audio: Relaxation Techniques, Mindful Meditations, Music and Soundscapes | 63 |
| Video: Animations and Video Programme Intros | 2 |
| Quiz: Test Learning | 10 |
| Game: Working Memory Game | 1 |

### Table 2: Application programme names and descriptions

| Programme Name | Description |
| --- | --- |
| Master Relaxation | Programme to develop skill of diaphragmatic breathing and progressive muscle relaxation |
| 5 Steps to Being Mindful | Teaches basics of mindfulness |
| Challenge Your Thoughts | CBT journaling and reflection. Learn about cognitive distortions and techniques to balance thinking |
| Constructive Worry | CBT-based Constructive worry and postponing worry (worry box) |
| Take Steps to More Positive Thinking | Skill of gratitude journaling |
| First Steps to A Better Night’s Sleep | CBTi programme psychoeducation and sleep hygiene) |
| Take On Healthy Sleep Habits | CBTi sleep hygiene and routines |
| Change How You Think About Sleep | CBTi on thoughts about sleep and thinking traps |
| Gain Control of Your Sleep | CBTi sleep scheduling |
| Falling Asleep | Techniques and audios for falling asleep |
| Build Your Self-Esteem | CBT programme on automatic thoughts and self-esteem |
| Boost Your Confidence | CBT - identify strengths |
| Thoughtful Communication | Developing in skills on communication. Based on Rosenburg's Non-violent communication |
| Mind Your Body | Psychoeducation (learning and quizzes) on the relationship between physical and mental health. Nutrition and physical activity |
| Move More—Feel Better 1 | three programmes developed with sports England and UCL focussing on increasing physical activity - targets nonactive users |
| Move More—Feel Better 2 |  |
| Move More—Feel Better 3 |  |
| Digital Balance | psychoeducation and interactive content on decreasing phone usage |
| The Pleasure-Purpose Principle | programme developed with Paul Dolan LSE on balancing pleasurable and purposeful activities to find happiness |

### Table 3: Comparison of patients included and excluded from ITT population at baseline.

|  | |  | | | Included (n=894) | Not included (n=108) | P-value |
| --- | --- | --- | --- | --- | --- | --- | --- |
| Age years | |  | | |  |  | 0.291 |
| 18-29 |  | | |  | 120 (88.89%) | 15 (11.11%) |  |
| 30-39 | |  | | | 189 (91.30%) | 18 (8.70%) |  |
| 40-49 | |  | | | 260 (89.04%) | 32 (10.96%) |  |
| 50-59 | |  | | | 237 (86.50%) | 37 (13.50%) |  |
| 60+ | |  | | | 88 (93.62%) | 6 (6.38%) |  |
| Gender | |  | | |  |  |  |
| Female | |  | | | 754 (88.92%) | 94 (11.08%) | 0.634 |
| Male | |  | | | 135 (91.22%) | 13 (8.78%) |  |
| Prefer not to say | | |  | | 5 (83.33%) | 1 (16.67%) |  |
| Ethnicity^1^ | |  | | |  |  |  |
| White | |  | | | 817 (89.09%) | 100 (10.91%) | 0.646 |
| Black/African/Caribbean/Black British | | | | | 13 (81.25%) | 3 (18.75%) |  |
| Asian/Asian British | | | | | 39 (90.70%) | 4 (9.30%) |  |
| Mixed/Multiple racial and ethnic groups | | | | | 18 (94.74%) | 1 (5.26%) |  |
| Other racial and ethnic minority groups | | | | | 6 (100.00%) | 0 (0.00%) |  |
| Use of application | | | | |  |  |  |
| Yes | |  | | | 110 (90.16%) | 12 (9.84%) | 0.720 |
| No | |  | | | 784 (89.09%) | 96 (10.91%) |  |
| Receiving therapy | | | | |  |  |  |
| Yes | | | | | 61 (87.14%) | 9 (12.86%) | 0.561 |
| No | | | | | 833 (89.38%) | 99 (10.62%) |  |
| Using mental health medication | | | | |  |  |  |
| Yes | | | | | 227 (92.65%) | 18 (7.35%) | 0.046 |
| No | | | | | 667 (88.11%) | 90 (11.89%) |  |
| Occupational Role | | | | |  |  |  |
| Clinical | |  | | | 544 (88.03%) | 74 (11.97%) | 0.122 |
| Non-clinical | |  | | | 350 (91.15%) | 34 (8.85%) |  |
| Country of birth^2^ | | | | |  |  |  |
| United Kingdom | | | | | 778 (88.81%) | 98 (11.19%) | 0.156 |
| EU (not UK) | |  | | | 43 (97.73%) | 7 (9.09%) |  |
| Other | |  | | | 70 (90.91%) | 1 (2.27%) |  |

^1^Missing (n=1); ^2^Missing (n=5)

### Table 4: Primary and secondary outcome assessments completed at baseline, week 4 and week 8.

|  | Baseline | | | Week 4 | | | Week 8 | | | |
| --- | --- | --- | --- | --- | --- | --- | --- | --- | --- | --- |
|  | App (n=502) | Control (n=500) | Total (n=1002) | App (n=391) | Control (n=455) | Total (n=846) | App (n=375) | Control (n=434) | Total (n=809) |  |
| GHQ-12 n, mean (SD) | 502, 15.81 (6.12) | 500, 15.92 (6.01) | 1002, 15.87 (6.07) | 391, 13.47 (6.96) | 455, 14.68 (6.71) | 846, 14.12 (6.85) | 375, 13.30 (6.35) | 434, 14.63 (6.47) | 809, 14.01 (6.44) |  |
| BRS n, mean (SD) | 502, 3.14 (0.80) | 500, 3.10 (0.81) | 1002, 3.12 (0.80) | 372, 3.18 (0.83) | 443, 3.15 (0.82) | 815, 3.16 (0.82) | 374, 3.22 (0.80) | 432, 3.13 (0.82) | 806, 3.17 (0.81) |  |
| SWEMWBS n, mean (SD) | 500, 20.40 (3.65) | 497, 20.40 (3.37) | 997, 20.40 (3.51) | 369, 20.78 (4.13) | 443, 20.52 (3.67) | 812, 20.64 (3.88) | 370, 21.28 (4.17) | 431, 20.59 (3.94) | 801, 20.91 (4.06) |  |
| SPS-6 n, mean (SD) | 495, 20.10 (5.16) | 496, 20.00 (4.95) | 991, 20.05 (5.06) | 369, 20.62 (5.13) | 443, 20.28 (5.18) | 812, 20.44 (5.15) | 370, 20.95 (5.04) | 430, 20.38 (5.22) | 800, 20.44 (5.15) |  |
| GAD-7 |  |  |  |  |  |  |  |  |  |  |
| Minimal anxiety n (%) | 201 (40.04%) | 199 (39.80%) | 400 (39.92%) | 165 (38.82%) | 191 (40.72%) | 356 (39.82%) | 189 (44.47%) | 211 (44.99%) | 400 (44.74%) |  |
| Mild anxiety n (%) | 199 (39.64%) | 196 (39.20%) | 395 (39.42%) | 142 (33.41%) | 163 (34.75%) | 305 (34.12%) | 117 (27.53%) | 133 (28.36%) | 250 (27.96%) |  |
| Moderate anxiety n (%) | 65 (12.95%) | 71 (14.20%) | 136 (13.57%) | 41 (9.65%) | 51 (10.87%) | 92 (10.29%) | 45 (10.59%) | 51 (10.87%) | 96 (10.74%) |  |
| Severe anxiety n (%) | 35 (6.97%) | 32 (6.40%) | 67 (6.69%) | 22 (5.18%) | 38 (8.10%) | 60 (6.71%) | 22 (5.18%) | 37 (7.89%) | 59 (6.60%) |  |
| Missing n (%) | 2 (0.40%) | 2 (0.40%) | 4 (0.40%) | 55 (12.94%) | 26 (5.54%) | 81 (9.06%) | 52 (12.24%) | 37 (7.89%) | 89 (9.96%) |  |
| PHQ-9 |  |  |  |  |  |  |  |  |  |  |
| Minimal depression n (%) | 210 (41.83%) | 200 (40.00%) | 410 (40.92%) | 158 (37.18%) | 179 (38.17%) | 337 (37.70%) | 174 (40.94%) | 184 (39.23%) | 358 (40.04%) |  |
| Mild depression n (%) | 144 (28.69%) | 158 (31.60%) | 302 (30.14%) | 116 (27.29%) | 141 (30.06%) | 257 (28.75%) | 108 (25.41%) | 126 (26.87%) | 234 (26.17%) |  |
| Moderate depression n (%) | 83 (16.53%) | 75 (15.00%) | 158 (15.77%) | 61 (14.35%) | 66 (14.07%) | 127 (14.21%) | 49 (11.53%) | 68 (14.50%) | 117 (13.09%) |  |
| Moderately severe depression n (%) | 49 (9.76%) | 42 (8.40%) | 91 (9.08%) | 21 (4.94%) | 31 (6.61%) | 52 (5.82%) | 28 (6.59%) | 31 (6.61%) | 59 (6.60%) |  |
| Severe depression n (%) | 14 (2.79%) | 22 (4.40%) | 36 (3.59%) | 13 (3.06%) | 26 (5.54%) | 39 (4.36%) | 12 (2.82%) | 22 (4.69%) | 34 (3.80%) |  |
| Missing n (%) | 2 (0.40%) | 3 (0.60%) | 5 (0.50%) | 56 (13.18%) | 26 (5.54%) | 82 (9.17%) | 54 (12.71%) | 38 (8.10%) | 92 (10.29%) |  |
| WSAS |  |  |  |  |  |  |  |  |  |  |
| Subclinical n (%) | 232 (46.22%) | 243 (48.60%) | 475 (47.41%) | 173 (40.71%) | 204 (43.50%) | 377 (42.17%) | 201 (47.29%) | 216 (46.06%) | 417 (46.64%) |  |
| Significant functional impairment n (%) | 188 (37.45%) | 181 (36.20%) | 369 (36.83%) | 143 (33.65%) | 172 (36.67%) | 315 (35.23%) | 121 (28.47%) | 147 (31.34%) | 268 (29.98%) |  |
| Moderately severe psychopathology n (%) | 79 (15.74%) | 72 (14.40%) | 151 (15.07%) | 53 (12.47%) | 67 (14.29%) | 120 (13.42%) | 48 (11.29%) | 68 (14.50%) | 116 (12.98%) |  |
| Missing n (%) | 3 (0.60%) | 4 (0.80%) | 7 (0.70%) | 56 (13.18%) | 26 (5.54%) | 82 (9.17%) | 55 (12.94%) | 38 (8.10%) | 93 (10.40%) |  |
| MISS |  |  |  |  |  |  |  |  |  |  |
| Mild/Moderate insomnia n (%) | 301 (59.96%) | 303 (60.60%) | 604 (60.28%) | 256 (60.24%) | 277 (59.06%) | 533 (59.62%) | 267 (62.82%) | 264 (56.29%) | 531 (59.40%) |  |
| Severe insomnia n (%) | 194 (38.65%) | 193 (38.60%) | 387 (38.62%) | 113 (26.59%) | 166 (35.39%) | 279 (31.21%) | 103 (24.24%) | 167 (35.61%) | 270 (30.20%) |  |
| Missing n (%) | 7 (1.39%) | 4 (0.80%) | 11 (1.10%) | 56 (13.18%) | 26 (5.54%) | 82 (9.17%) | 55 (12.94%) | 38 (8.10%) | 93 (10.40%) |  |

#### Data are n, mean (SD) or n (%). Higher scores better for BRS, SWEMWBS, SPS-6.

### Table 5: Comparison of patients included and excluded from ITT population at baseline.

|  | Included (n=894) | | | Not included (n=108) | | | |
| --- | --- | --- | --- | --- | --- | --- | --- |
|  | App (n=425) | Control (n=469) | Total (n=894) | | App (n=77) | Control (n=31) | Total (n=108) |
| GHQ-12 n, mean (SD) | 425, 16.05 (6.14) | 469, 16.00 (5.94) | 894, 16.02 (6.03) | | 77, 14.47 (5.89) | 31, 14.81 (7.01) | 108, 14.56 (6.20) |
| BRS n, mean (SD) | 425, 3.13 (0.81) | 469, 3.09 (0.82) | 894, 3.11 (0.81) | | 77, 3.19 (0.69) | 31, 3.13 (0.79) | 108, 3.17 (0.71) |
| SWEMWBS n, mean (SD) | 425, 20.37 (3.68) | 468, 20.43 (3.41) | 893, 20.40 (3.54) | | 75, 20.61 (3.49) | 29, 20.00 (2.76) | 104, 20.44 (3.30) |
| SPS-6 n, mean (SD) | 424, 20.11 (5.18) | 467, 19.99 (4.96) | 891, 20.05 (5.06) | | 71, 20.07 (5.10) | 29, 20.07 (4.99) | 100, 20.07 (5.05) |
| GAD-7 |  |  |  | |  |  |  |
| Minimal anxiety n (%) | 168 (39.53%) | 188 (40.09%) | 356 (39.82%) | | 33 (42.86%) | 11 (35.48%) | 44 (40.74%) |
| Mild anxiety n (%) | 167 (39.29%) | 184 (39.23%) | 351 (39.26%) | | 32 (41.56%) | 12 (38.71%) | 44 (40.74%) |
| Moderate anxiety n (%) | 57 (13.41%) | 67 (14.29%) | 124 (13.87%) | | 8 (10.39%) | 4 (12.90%) | 12 (11.11%) |
| Severe anxiety n (%) | 33 (7.76%) | 30 (6.40%) | 63 (7.05%) | | 2 (2.60%) | 2 (6.45%) | 4 (3.70%) |
| Missing n (%) | 0 (0.00%) | 0 (0.00%) | 0 (0.00%) | | 2 (2.60%) | 2 (6.45%) | 4 (3.70%) |
| PHQ-9 |  |  |  | |  |  |  |
| Minimal depression n (%) | 171 (40.24%) | 187 (39.87%) | 358 (40.04%) | | 39 (50.65%) | 13 (41.94%) | 52 (48.15%) |
| Mild depression n (%) | 123 (28.94%) | 151 (32.20%) | 274 (30.65%) | | 21 (27.27%) | 7 (22.58%) | 28 (25.93%) |
| Moderate depression n (%) | 75 (17.65%) | 70 (14.93%) | 145 (16.22%) | | 8 (10.39%) | 5 (16.13%) | 13 (12.04%) |
| Moderately severe depression n (%) | 43 (10.12%) | 40 (8.53%) | 83 (9.28%) | | 6 (7.79%) | 2 (6.45%) | 8 (7.41%) |
| Severe depression n (%) | 13 (3.06%) | 20 (4.26%) | 33 (3.69%) | | 1 (1.30%) | 2 (6.45%) | 3 (2.78%) |
| Missing n (%) | 0 (0.00%) | 1 (0.21%) | 1 (0.11%) | | 2 (2.60%) | 2 (6.45%) | 4 (3.70%) |
| WSAS |  |  |  | |  |  |  |
| Subclinical n (%) | 198 (46.59%) | 225 (47.97%) | 423 (47.32%) | | 34 (44.16%) | 18 (58.06%) | 52 (48.15%) |
| Significant functional impairment n (%) | 152 (35.76%) | 174 (37.10%) | 326 (36.47%) | | 36 (46.75%) | 7 (22.58%) | 43 (39.81%) |
| Moderately severe psychopathology n (%) | 75 (17.65%) | 68 (14.50%) | 143 (16.00%) | | 4 (5.19%) | 4 (12.90%) | 8 (7.41%) |
| Missing n (%) | 0 (0.00%) | 1 (0.21%) | 1 (0.11%) | | 3 (3.90%) | 2 (6.45%) | 5 (4.63%) |
| MISS |  |  |  | |  |  |  |
| Mild/Moderate insomnia n (%) | 256 (60.24%) | 287 (61.19%) | 543 (60.74%) | | 45 (58.44%) | 16 (51.61%) | 61 (56.48%) |
| Severe insomnia n (%) | 168 (39.53%) | 180 (38.38%) | 348 (38.93%) | | 26 (33.77%) | 13 (41.94%) | 39 (36.11%) |
| Missing n (%) | 1 (0.23%) | 2 (0.43%) | 3 (0.34%) | | 6 (7.79%) | 2 (6.45%) | 8 (7.41%) |

### Table 6. Average amount of time (minutes) spent using the application overall and weekly from those who downloaded the application.

|  | N | Mean (SD) | Median (LQ-UQ) | Range |
| --- | --- | --- | --- | --- |
| Overall | 379 | 100.37 (149.29) | 46.82 (16.62-129.81) | 0.67-1011.70 |
| Week 1 | 379 | 32.39 (41.14) | 17.98 (6.25-40.12) | 0.52-304.25 |
| Week 2 | 379 | 17.02 (29.34) | 4.50 (0.00-22.57) | 0.00-190.98 |
| Week 3 | 379 | 13.66 (27.61) | 1.10 (0.00-16.62) | 0.00-230.00 |
| Week 4 | 379 | 11.92 (22.92) | 1.05 (0.00-14.08) | 0.00-139.95 |
| Week 5 | 379 | 9.65 (22.28) | 0.02 (0.00-10.25) | 0.00-168.97 |
| Week 6 | 379 | 6.93 (16.69) | 0.00 (0.00-4.58) | 0.00-132.63 |
| Week 7 | 379 | 4.69 (12.83) | 0.00 (0.00-1.08) | 0.00-106.47 |
| Week 8 | 379 | 4.10 (12.85) | 0.00 (0.00-1.12) | 0.00-107.80 |

### Table 7. Average amount of time (minutes) spent using the application overall and weekly from those who started one activity.

|  | N | Mean (SD) | Median (LQ-UQ) | Range |
| --- | --- | --- | --- | --- |
| Overall | 357 | 106.32 (151.83) | 51.62 (19.65-131.78) | 1.07-1011.70 |
| Week 1 | 326 | 36.99 (42.57) | 22.25 (9.76-46.43) | 0.90-304.25 |
| Week 2 | 230 | 27.99 (33.36) | 16.34 (6.63-34.80) | 0.28-190.98 |
| Week 3 | 195 | 26.28 (33.96) | 15.78 (6.07-30.47) | 0.17-230.00 |
| Week 4 | 188 | 23.49 (28.09) | 14.01 (5.40-29.59) | 0.03-139.95 |
| Week 5 | 155 | 23.27 (30.01) | 12.07 (5.92-27.85) | 0.25-168.97 |
| Week 6 | 122 | 21.24 (23.72) | 12.67 (5.70-26.59) | 0.17-132.63 |
| Week 7 | 98 | 17.73 (20.08) | 11.69 (3.87-21.12) | 0.17-106.47 |
| Week 8 | 92 | 16.57 (21.85) | 6.67 (2.69-21.65) | 0.20-107.80 |

### Table 8: Per Protocol Population definitions to compliance, and results for the sensitivity analysis for the primary outcome: GHQ-12

|  |  |  | **Adjusted Mean Difference (aMD)*** | | | |
| --- | --- | --- | --- | --- | --- | --- |
| **Outcome** | **PPP*** | **N** | **Mean difference** | **Lower CI** | **Upper CI** | **P value** |
| **GHQ-12** | **Download** | **700** | **-1.39** | **-2.08** | **-0.70** | **<0.001** |
|  | **Two activities in week 1 and 2** | **610** | **-1.21** | **-1.98** | **-0.43** | **0.002** |
|  | **Four activities in week 1 and 2** | **574** | **-1.09** | **-1.92** | **-0.25** | **0.01** |
|  | **One programme in week 1 and 2** | **521** | **-1.12** | **-2.08** | **-0.17** | **0.02** |
|  | **Twenty-nine activities** **overall** | **591** | **-1.50** | **-2.33** | **-0.68** | **<0.001** |

*Adjusted for: baseline outcome, age, sex, ethnicity, occupational role (clinical or non-clinical), use of other mental health application (yes or no), use of mental health/wellbeing medication (yes or no), use of psychological or talking therapy (yes or no), and 4-week and 8-week assessments.

One participant dropped due to missing ethnicity.
