# Supplementary material for: Multicentre, England-wide randomised controlled trial of the ‘Foundations’ smartphone application in improving mental health and well-being in a healthcare worker population
Source: Br J Psychiatry. 2023 Feb;222(2):58–66. doi: 10.1192/bjp.2022.103 (PMC10895508; doi:10.1192/bjp.2022.103)
Supplement: Supplementary file 1 [file S0007125022001039sup.zip › S0007125022001039sup001.pdf]

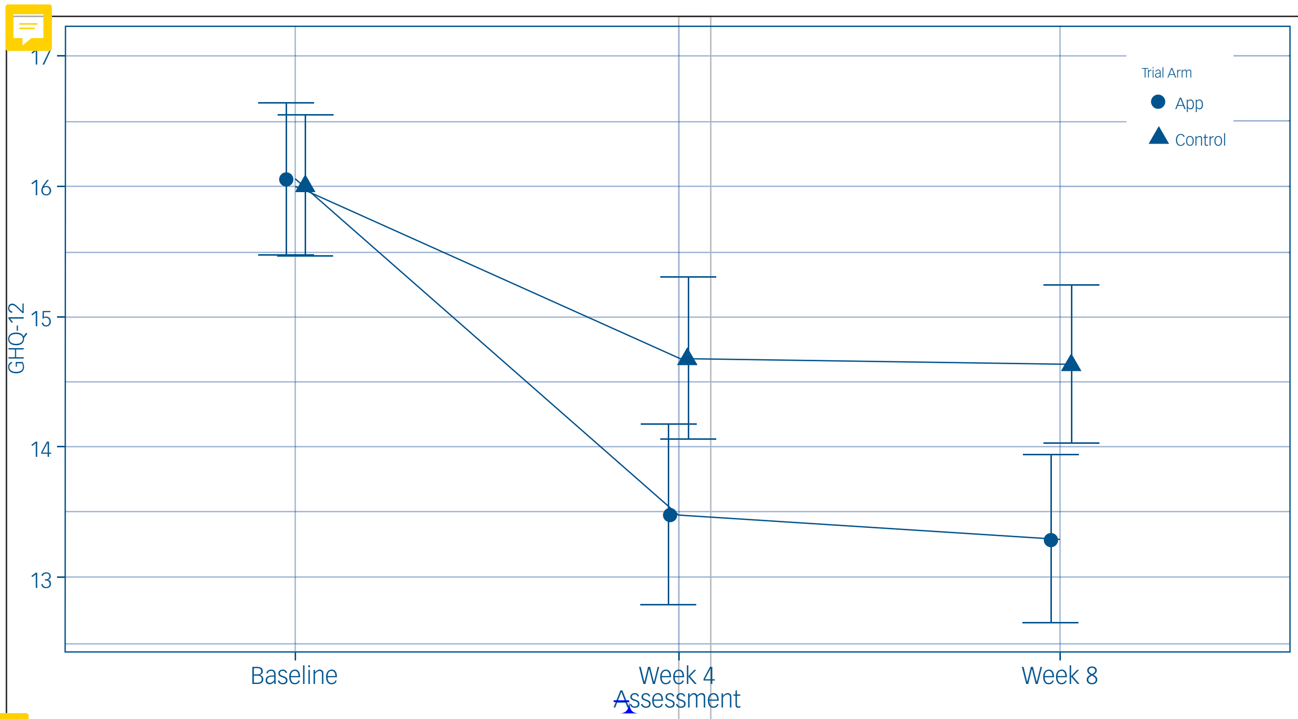

FileName : \S0007125022001039_fig1.eps, Size : (40.12 x 21.47)pica, UserId : jrnlgfx

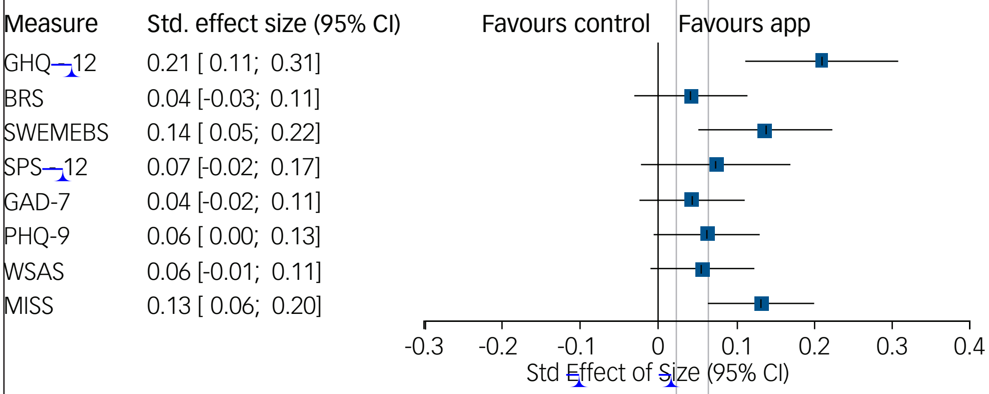

FileName : \S0007125022001039_fig2.eps, Size : (30.66 x 11.70)pica, UserId : jrnlgfx

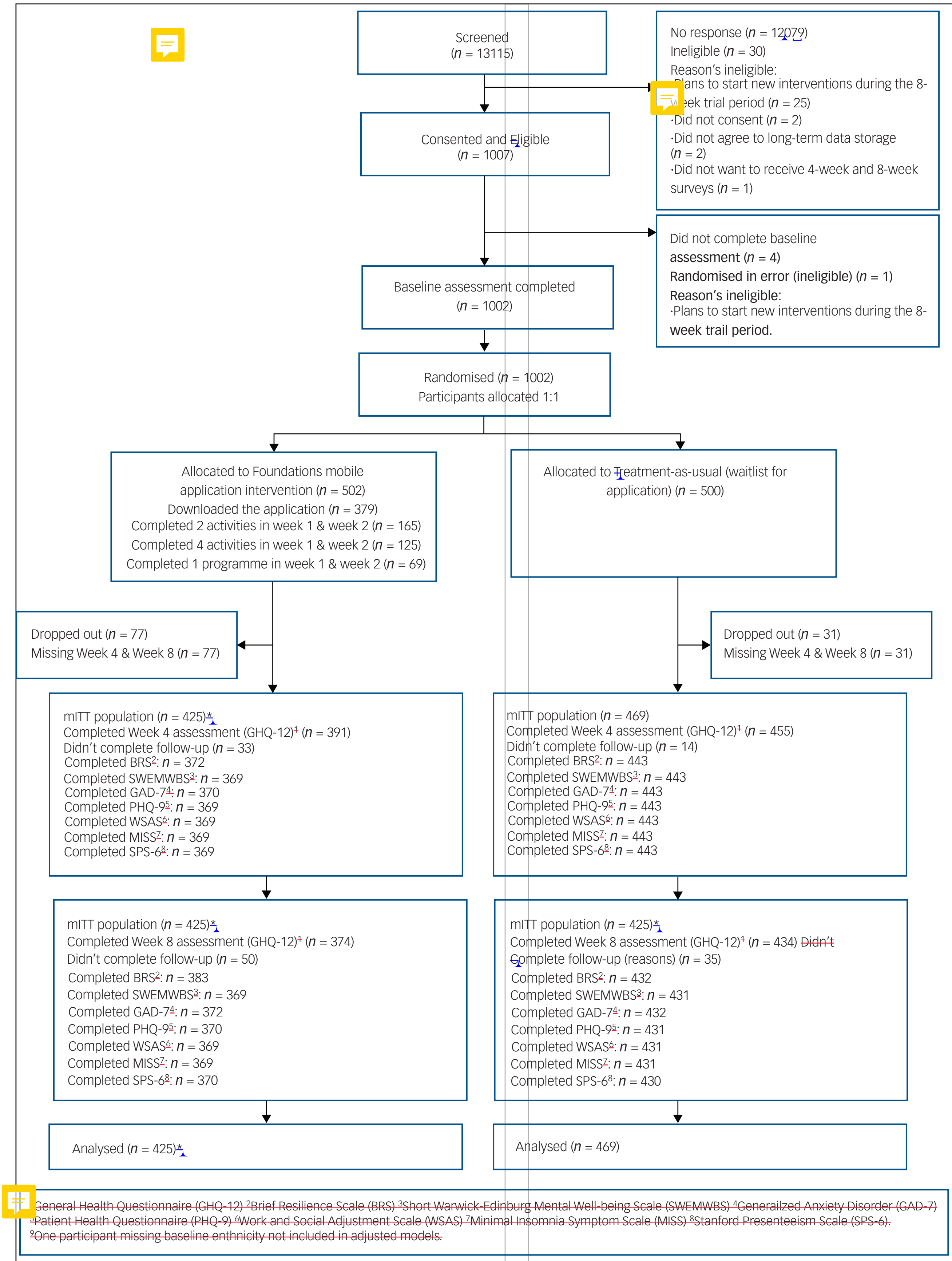

FileName : \S0007125022001039_inline1.eps, Size : (40.06 x 53.55)pica, UserId : jrnlgfx
