# Supplementary material for: Multicentre, England-wide randomised controlled trial of the ‘Foundations’ smartphone application in improving mental health and well-being in a healthcare worker population
Source: Br J Psychiatry. 2023 Feb;222(2):58–66. doi: 10.1192/bjp.2022.103 (PMC10895508; doi:10.1192/bjp.2022.103)
Supplement: Supplementary file 1 [file S0007125022001039sup.zip › S0007125022001039sup002.pdf]

STUDY PROTOCOL:

Overall Study Name: NHS CHECK - Health & Experiences of staff working at NHS Trusts and Nightingale Hospitals.

Embedded RCT Short Title: Smartphone app-based mental health and wellbeing intervention (Foundations App) for healthcare workers during Covid-19.

Embedded RCT Full Title: Effectiveness of a smartphone app-based intervention (Foundations App) in improving stress, resilience, wellbeing and mental health outcomes in a high-risk healthcare worker population during Covid-19: a randomised controlled parallel group trial.

Contents

STUDY PROTOCOL: 1
Administrative Information 4
1. Study Title 4
2. Trial Registration 4
3. Protocol version 4
4. Funding 4
5. Roles and Responsibilities 4
Introduction 8
6. Background and Rationale 8
7. Trial Design 8
Methods: Participants, Interventions & Outcomes 9
8. Study Setting 9
9. Eligibility Criteria 9
10. Interventions 10
11. Baseline and outcome measures 111
Table of measures and timings 112
12. Participant Timeline: 123
13. Sample Size: 13
14. Recruitment: 13
Assignment of Interventions 14
15. Allocation: 14
16. Blinding: 14
Data Collection, Management and Analysis 14
17. Data Collection Methods: 14
18. Data Management 15
19. Statistical Methods 15
Monitoring 16
20. Data Monitoring: 17
21. Harms: 17
Ethics and Dissemination 20
22. Research Ethics Approval 19
23. Protocol Amendments 19
24. Consent 19
25. Confidentiality 19
26. Declaration of Interests 19

27. Access to data 19
28. Dissemination Policy 19
Appendices 19
39. Informed consent materials 19
References 19

Administrative Information

1. Study Title: NHS CHECK: Health & Experiences of staff working at NHS Trusts and Nightingale Hospitals

2. Trial Registration

ISRCTN:	ISRCTN18395399 (NHS CHECK Study)		
REC Number:	20/HRA/2107		
UKCRN Number:	CPMS46176		
Protocol Version Number:	2	Date:	24/05/2021

3. Protocol version

Protocol version 2; 24.05.2021

4. Funding

This randomised control trial is funded by Koa Health.

5. Roles and Responsibilities

(Co) Sponsor(s)

Name:	King's College London
Address:	James Clerk Maxwell Building<br>57 Waterloo Road<br>SE1 8WA
Telephone:	02071886206
Email:	Thomas.foulkes@kcl.ac.uk<br>R&D@gstt.nhs.uk

Chief Investigators

Name:	Professor Simon Wessely
Address:	King's College London, Psychological Medicine, Institute of Psychiatry, Psychology and Neuroscience, Weston Education Centre, 3rd floor, 10 Cutcombe Road, SE5 9AF
Email:	simon.wessely@kcl.ac.uk

Name:	Dr. Sam Gnanapragasam
Address:	King's College London, Psychological Medicine, Institute of Psychiatry, Psychology and Neuroscience, De Crespigny Park, London, SE5 8AF
Email:	sam.gnanapragasam@kcl.ac.uk

Co-Investigator(s), Statistician, Therapy Service, Laboratories etc

Name:	Rose Tinch-Taylor
Position/ Role:	Trial statistician
Address:	Institute of Psychiatry, Psychology & Neuroscience, De Crespigny Park, London, SE5 8AF
Email:	Rose.tinch-taylor@kcl.ac.uk

Name:	Dr Ben Carter
Position/ Role:	Senior Lecturer in Biostatistics<br>KCTU Mental Health Statistics Group Lead
Address:	Department of Biostatistics, Institute of Psychiatry, Psychology & Neuroscience, De Crespigny Park, London, SE5 8AF
Email:	Ben.carter@kcl.ac.uk

Name:	Rupa Bhundia
Position/ Role:	Project Manager

Address:	Institute of Psychiatry, Psychology & Neuroscience, De Crespigny Park, London, SE5 8AF
Email:	rupa.bhundia@kcl.ac.uk

Name:	Professor Neil Greenberg
Position/ Role:	Professor of Defence Mental Health<br>Consultant occupational and forensic psychiatrist
Address:	King's Centre for Military Health Research, King's College London, Institute of Psychiatry, Psychology and Neuroscience, Weston Education Centre, 3rd floor, 10 Cutcombe Road, SE5 9AF
Email:	neil.greenberg@kcl.ac.uk

Name:	Dr. Sharon Stevelink
Position/ Role:	Lecturer in Epidemiology
Address:	King's College London, Psychological Medicine, Institute of Psychiatry, Psychology and Neuroscience, De Crespigny Park, London, SE5 8AF
Email:	sharon.stevelink@kcl.ac.uk

Name:	Dr. Ira Madan
Position/ Role:	Director, Occupational Health, Guys and St Thomas' NHS Foundation Trust
Address:	St Thomas' Hospital, Westminster Bridge Road , London, SE1 7EH
Email:	ira.madan@gsst.nhs.uk

Name:	Dr. Danielle Lamb
Position/ Role:	Senior Research Fellow, Institute of Epidemiology & Health, University College London
Address:	Applied Health Research, Institute of Epidemiology & Health, Faculty of Pop Health Sciences,
Email:	d.lamb@ucl.ac.uk

Name:	Dr. Hannah Scott

Position/ Role:	Post Doctoral Researcher
Address:	King's College London, Psychological Medicine, Institute of Psychiatry, Psychology and Neuroscience, De Crespigny Park, London, SE5 8AF
Email:	hannah.r.scott@kcl.ac.uk

Name:	Emilia Souliou
Position/ Role:	Research Assistant
Address:	King's College London, Psychological Medicine, Institute of Psychiatry, Psychology and Neuroscience, De Crespigny Park, London, SE5 8AF
Email:	emilia.souliou@kcl.ac.uk

Name:	Siobhan Hegarty
Position/ Role:	Research Assistant
Address:	King's College London, Psychological Medicine, Institute of Psychiatry, Psychology and Neuroscience, De Crespigny Park, London, SE5 8AF
Email:	siobhan.hegarty@kcl.ac.uk

Name:	Danny Weston
Position/ Role:	Data Manager
Address:	King's College London, Psychological Medicine, Institute of Psychiatry, Psychology and Neuroscience, De Crespigny Park, London, SE5 8AF
Email:	danny.l.weston@kcl.ac.uk

Trial Management Group (TMG)

The TMG comprises:

Rose Tinch-Taylor – trial statistician
Dr Ben Carter – statistical and trials expertise
Rupa Bhundia – project manager
Professor Simon Wessely – co-chief investigator
Dr Sam Gnanapragasam – co-chief investigator
Professor Neil Greenberg – co-investigator
Dr Sharon Stevelink – co-investigator
Dr Danielle Lamb – senior research fellow
Dr Hannah Scott – post doctoral researcher
Siobhan Hegarty – research assistant
Emilia Souliou – research assistant
Danny Weston – data manager

Introduction

6. Background and Rationale

Foundations is a mental wellbeing app with interactive activities and programmes designed to build resilience, manage stress and improve sleep. It includes psychoeducational content aimed at promoting well-being based on cognitive behavioural therapy (CBT), mindfulness-based CBT, insomnia targeted CBT, relaxation techniques and positive psychology. It includes a mixture of standalone activities and programmes. Activities are delivered through a variety of formats including journaling, learning articles and slides, audios and quizzes; a programme comprises a sequenced set of activities.

Population of interest

The study population is defined as: all staff working within 18 NHS Trusts across England (listed below under section 8. Study Settings), including medical, nursing, midwifery, allied health professionals, support, administrative and management staff, as well as students who volunteered or were fast-tracked into clinical roles during the COVID-19 pandemic.

Aims and Objectives

To evaluate whether the use of Foundations mobile application impacts stress, wellbeing, anxiety, depression, functioning, resilience and sleep in a real-world, high-risk healthcare worker cohort.

7. Trial Design

This will be a parallel group design, unblinded randomised controlled trial of the Foundations app versus or waiting list control (1:1 ratio) with measures taken at baseline, 4 weeks and 8.

Participants will be randomised individually with equal allocation to the two arms, stratified by site and occupational role (clinical or nonclinical), using a random permuted block design. This will be implemented using an independent online system based at the King's Clinical Trials Unit (King's CTU) based at King's College London.

Methods: Participants, Interventions & Outcomes

8. Study Setting

The following 18 NHS Trusts have been selected to include a mixture of urban and rural settings:

Avon and Wiltshire Mental Health Mental Health Partnership Trust
Cambridge University Hospitals NHS Foundation Trust
Cambridgeshire and Peterborough NHS Foundation Trust
Cornwall Partnership NHS Foundation Trust
Devon Partnership NHS Foundation Trust
East Suffolk and North Essex NHS Foundation Trust
Gloucestershire Hospitals NHS Foundation Trust
Guys and St Thomas' NHS Foundation Trust
King's College Hospital NHS Foundation Trust
Lancashire and South Cumbria NHS Foundation Trust
Norfolk and Norwich University Hospitals Foundation Trust
Nottinghamshire Healthcare NHS Foundation Trust
Royal Papworth Hospital NHS Foundation Trust
Sheffield Health and Social Care NHS Foundation Trust
South London and Maudsley NHS Foundation Trust
Tees Esk and Wear Valleys NHS Foundation Trust
University Hospitals of Derby and Burton NHS Foundation Trust
University Hospitals of Leicester NHS Foundation Trust

9. Eligibility Criteria

Inclusion:

1. Be aged 18 and over.
2. Be an NHS-affiliated member of staff, working at, or with, the participating eighteen NHS CHECK sites.
3. Completed baseline survey with NHS CHECK Cohort study and have consented to be contacted for further research as part of study.
4. Be able to give informed consent to take part in research.
5. Be able to understand and communicate in English.
6. Have access to the internet to complete the surveys.
7. Have access to an email address to facilitate application registration and receive survey links.
8. Own a smartphone with access to the Apple and Google Application Stores.

Exclusion:

1. Plans to start new interventions after randomisation during eight week trial period (e.g. apps, psychological therapies and pharmacological therapies).

10. Interventions

Interventions

Eligible participants will be randomised to receive the Foundations app or be part of the control arm (waitlist for the app). Randomisation will be carried out by the KCL CTU (King’s College London Clinical Trials Unit).
Participants in the wait-list control arm will continue treatment as usual.

Participants in the intervention arm will be asked to use Foundation App, provided by Koa Health, in addition to continuing treatment as usual.

All participants will be asked to complete surveys at baseline, week 4 and week 8 comprising the measures detailed below. All participants receive the same emails and text messages to complete the 4 and 8 week follow-up surveys. This will be sent three times in week 4 and week 8.

Participant withdrawal

Participants can withdraw by stopping completion of the online surveys. Participants can also email the study team using the dedicated study email address (nhscheck@kcl.ac.uk) to indicate they are no longer willing to participate in the study. We will then ensure that their email is removed from the email distribution lists. We aim to publish non-identifiable data in brief summaries within weeks of data collection. Therefore, withdrawal of the data from published summaries/papers will not be feasible. This is clearly explained in the information sheets.

Adherence

Usage data will be used to assess participant engagement, adherence to the intervention. Data will be collected on how the Foundations app is used (i.e. frequency, activities undertaken) and the text that users input. This will be further defined in the Statistical Analysis Plan (SAP), prior to the database lock.

Participants in the intervention arm will be contacted with scheduled emails twice a week from the Foundations App in accordance with usual-application use; participants may also opt in within the app to receive push notifications 2-3 times a week to encourage application use. Based upon adherence/use in week one and two, further engagement may occur such as study team contacting participants via email and text messages to continue using the application.

Concomitant care and interventions

Participants will be informed that once recruited they may continue with any current interventions but should not start any new ones.

11. Baseline and outcome measures

Please see table below for timings.

Primary outcome measure:

1. The GHQ-12 is a 12-item scale which screens for general (non-psychotic) psychiatric morbidity, (Goldberg et al., 1992).

Secondary outcome measures:

2. The Brief Resilience Scale (BRS) to measure resilience is a 6-item participant self-report measure, which assesses an individual's ability to bounce back or recover from stress. (Smith et al., 2008).
3. The GAD-7 is a well validated and widely used 7-item measure of anxiety. A change of 4 or more on the GAD has been found to be clinically significant across anxiety disorders. (Spitzer et al., 2006).
4. The PHQ-9 is the 9-item depression module from the full PHQ. There is strong evidence for the validity of the PHQ-9 as a brief measure of depression severity. (Kroenke et al., 2001).
5. The Warwick-Edinburgh Mental Well-being Scale (WEMWBS) is a 7-item measure to enable the monitoring of subjective well-being and psychological functioning compared to the general population (all items are worded positively and address aspects of positive mental health) (Tennant et al., 2007).
6. The 5-item Work and Social Adjustment Scale (WSAS) (Mundt et al., 2002).
7. The 6-item Stanford Presenteeism Scale (SPS-6) (Koopman et al., 2002).
8. The Minimal Insomnia Symptom Scale (MISS) is a 3-item scale which provides a brief measure of sleeping difficulties (Broman et al., 2008).

Alongside the outcome measures listed above, data will be collected via the NHS baseline survey to include:

9. Current Psychological & Pharmacological Support (CPPS), including use of psychotropic medication, psychological therapies and mobile wellbeing apps.
10. COVID-19 stressors that have significantly impacted participants in the prior month.

Demographic information will be gathered from existing NHS CHECK surveys that participants completed.

11. Demographic data including sex, occupation, ethnicity, country of birth.

Table 1. Measures and timings

Measure	Existing NHS CHECK Survey	Baseline survey completion (pre-randomisation)	Week 4 survey completion	Week 8 survey completion
1. GHQ-12		X	X	X

2. BRS		X	X	X
3. GAD-7		X	X	X
4. PHQ-9		X	X	X
5. WEMWBS		X	X	X
6. WSAS		X	X	X
7. SPS-6		X	X	X
8. MISS		X	X	X
9. CPPS		X	X	X
10. Stressors		X	X	X
11. Demographic	X			

(For key, see above; numbers correspond to above list)

12. Participant Timeline:

Figure 1.

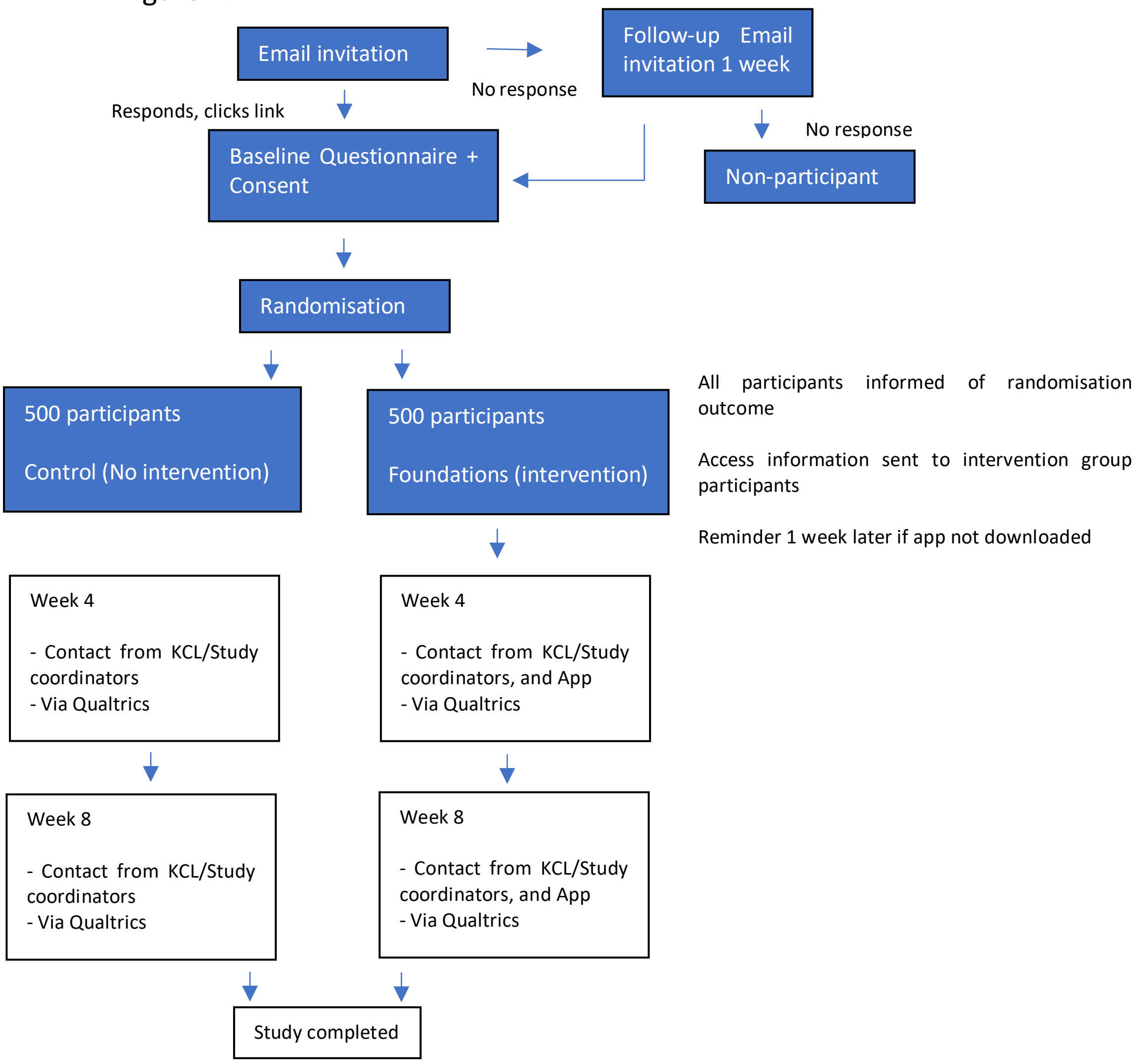

13. Sample Size:

This study requires 1000 participants with 500 in each trial arm. This sample size calculation is based on 80% power, 5% significance and a standardized effect size of 0.3. We also inflated the requirement to account for 50% retention at week 4.

14. Recruitment:

NHS CHECK cohort study participants from 18 NHS Trusts who have agreed to be contacted for further research studies will be invited to take-part via email. This email will include a link to the participant information sheet, which includes: an explanation of the study, invitation to take-part, link to participant information sheet, link to frequently asked questions and link to baseline survey (with consent included therein). For those who do not respond to the initial survey invitation, one follow-up email to be sent. Should there be non-response to this email, those invited to be deemed as 'non-participants'.

A specific study email (nhscheck@kcl.ac.uk) has been set up in case participants have any questions. Participant information will be provided on the website reinforcing that participation is entirely voluntary and no study data will be available in any identifiable format to anyone outside of the immediate research team such that those in authority in their employing organisations will not have access to their data.

Once participants have read the information sheet, consented to take-part and completed baseline survey, they will be checked against the inclusion/exclusion criteria. Suitable participants must have provided consent to be in the NHS CHECK Study and sharing their data with Koa Health.

Following randomisation, NHS CHECK to request Koa Health to white-list email addresses for intervention arm. NHS CHECK to inform participants of their randomisation outcome, and for those in the intervention group, to send participants App access/log-in details. The point at which this allocation email is sent to participants will count at day 0, for the timing of week 4 and week 8 follow-up surveys. Participants in the intervention group will be asked to download from the Google/Apple Appstore and register. Reminder 1 week later if app not downloaded (information to be provided by Koa Health).

At the end of the eight week trial period, wait-list control participants will be given full app access. They will be contacted by NHS CHECK and sent App access/log-in details.

All participants receive the same emails and text messages to complete the four and eight week follow-up surveys. This will be sent three times in week four and week eight. Those who use the app will also receive push notifications, up to three times in the week.

Financial Incentives:

- Vouchers will be emailed to participants to thank them for their participation.
- Participants will receive a remuneration after the final time point (8 weeks).
- If only the baseline survey is completed, participants will receive a £5 voucher.

- If two of the three surveys are completed (baseline and week 4 or baseline and week 8) participants will receive a £15 voucher.
- If participants complete all surveys (baseline, week 4 and week 8) they will receive a £25 voucher.

Assignment of Interventions

15. Allocation:

Participants will be allocated to the intervention group (receive the Foundations App) or control (waitlist for the app) at a ratio 1:1 via an independent online system at the King's Clinical Trials Unit (King's CTU) based at King's College London.

Randomisation

The sequence will be generated using a varying permuted block design (2 and 4 units) stratified by hospital trust and occupational role (clinical or non-clinical). The sequence will be concealed from the investigators including the Chief investigators and trial statistician. All study personnel and participants are blinded to the treatment allocation. Participants will be randomised via the online, central randomisation service based at King's CTU.

Implementation:

King's CTU will provide an online randomisation system that the research team will log onto then contact the enrolled users.

16. Blinding:

The participants and researchers will know of the allocation; it is not possible to blind participants due to the nature of the intervention. The senior statistician and chief investigators will be blind to allocation until the end of the trial when there is a planned unblinding. The trial statistician will be pseudo blind.

Data Collection, Management and Analysis

17. Data Collection Methods:

KCL will collect contact information (email address and contact telephone number) during the consent process.

Source demographic data will be gathered from the existing NHS CHECK cohort study baseline survey.

Questionnaire data will be collected via Qualtrics, with outcome measures including GHQ12, BRS, GAD7, PHQ9, WEMWBS, WSAS, SPS-6 and MISS as well as information about CPPS and current COVID-19 related stressors. These outcome measures will be further assessed at 4 and 8 weeks (see table above).

Koa Health will also collect information related to how the Foundations app is used (i.e. frequency, activities undertaken, etc.), and text that users input. Usage data will be shared with KCL CTU to investigate the interaction of engagement and efficacy.

Participants use Foundations according to the Foundations terms and conditions and the Foundations privacy policy. For Foundations users who participate in the KCL research, and who provide the additional consent to do so, Koa will share the data collected in Foundations that is described in the study privacy policy for the research purpose described in this document.

Participants are required to use their email address, and to provide consent to data sharing to Koa Health. This allows access to the app, linkage of the data collected in Foundations to the data collected in questionnaires and surveys, and research on the joined dataset.

Promotion of participant retention – data collection from dropout

Participants will receive an incentive (£25, £15 or £5) for the additional burden incurred from completing assessments at the final outcome point.

18. Data Management

A data impact assessment will be conducted, and the study will be registered on the King's Data Protection Register.

All information collected will be managed in the strictest confidence. Online survey responses will be collected using Qualtrics survey software which will be hosted by King's College London, on servers located in the UK. Data required for analysis will be downloaded in pseudonymised form and stored on KCL OneDrive. Any interactions with Qualtrics will be logged and audited by a designated researcher. We will use a valid Data Protection and Security Toolkit from the King's Centre for Military Health Research at King's College London and will follow this Toolkit during rollout (EE133874-MHR / Revision Date: 10/04/2020).

19. Statistical Methods

Analysis of Primary Outcome

Estimates of differences between the intervention and control group and their associated 95% confidence intervals will be reported. The significance level will be 5% (two-sided), using Stata version 16.

A linear mixed model will be used to estimate the difference in mean GHQ-12 score at 4 and 8 weeks between arms. Linear mixed modelling utilises all available information leading to more precise estimates of the intervention effect. This technique will allow the simultaneous modelling of the repeated outcome time points.

In such models the outcome variable measured during follow-up will feature as the dependent variable. Independent variables include the fixed effects of: trial arm, time (4 week or 8 week), baseline GHQ-12 score, age, sex, occupational role (clinical or non-clinical),

main hospital type (acute or mental health), use of other mental health app (yes or no), an increase in care (medication or psychological therapy); the random effects of site and participant.

Analysis of Secondary Outcomes

For the continuous secondary outcomes similar methodology to the primary outcome analysis. Fixed effects will adjust for baseline values of the outcome, trial arm, time (4 week or 8 week), age, sex, occupational role (clinical or non-clinical), main hospital type (acute or mental health), use of other mental health app (yes or no), recent change in medication and recent change in psychological therapy. Random effects of site and participant will be used.

Population under investigation and missing data

The primary and secondary outcome analyses will use a modified intention-to-treat population (ITT) unless otherwise specified. The per protocol population (PPP) will exclude patients defined as protocol violators (eg those that fail to have fidelity).

We anticipate negligible missing baseline covariate data, and this will be explored and may be imputed. Missing item follow up data within instruments will be pro-rata mean imputed if a minimum of 75% completion rate within instrument. Missing instrument data will be explored to assess the missing at random assumption.

Subgroups

The following subgroups will be explored: age; gender; occupation.

Parametric Assumption Checks

Model assumptions will be assess that the residuals are identically, independently, normally distributed with a zero mean and constant variance. Transformations will be considered where data may not be normally distributed.

Sensitivity analyses

A sensitivity will be carried out to assess the robustness of assumptions. This will be further defined in the SAP, for example a sensitivity analysis will be carried out on the primary outcome using the PPP.

Data Checking

Data queries will be carried out in the run up to database lock, these will assess the data completeness and quality for data used within the primary and secondary analyses.

CTU Quality Management Processes

KCTU Statistics Standard Operating Procedures will be followed, or a filenote will be signed by the trial statistician and stored within the Trial Master File by the Trial Manager.

Monitoring

20. Data Monitoring:

A trial steering group will be established to examine the clinical progress and study results. The conduct of the research will be reviewed by the research team at regular weekly meetings.

Trial steering group:
2x clinicians
1 statistician
1x expert by experience

21. Harms:

Adverse Effects

The Medicines for Human Use (Clinical Trials) Regulations 2004 and Amended Regulations 2006 gives the following definitions:

Adverse Event (AE):

- Any untoward medical occurrence in a subject to whom a medicinal product has been administered including occurrences which are not necessarily caused by or related to that product.

Adverse Reaction (AR):

- Any untoward and unintended response in a subject to an investigational medicinal product which is related to any dose administered to that subject.

Unexpected Adverse Reaction (UAR):

- An adverse reaction the nature and severity of which is not consistent with the information about the medicinal product in question set out in: The summary of product characteristics (SmPC) for Sativex®

Serious adverse Event (SAE), Serious Adverse Reaction (SAR) or Unexpected Serious Adverse Reaction (USAR):

- Any adverse event, adverse reaction or unexpected adverse reaction, respectively, that
    - Results in death;
    - Is life-threatening.
    - Required hospitalisation or prolongation of existing hospitalisation;
    - Results in persistent or significant disability or incapacity;
    - Consists of a congenital anomaly or birth defect.

Whilst there is no obligation to report a serious adverse event (SAE) to the Medicines and Healthcare Products Regulatory Agency (MHRA) as there is no medical intervention in this study, the NHS CHECK team is required to report any SAE that occurs, which is deemed related to research procedures and unexpected to the main Research Ethics Committee (REC).

The NHS Check team (Trial manager) will report to the main REC if the SAE occurred AND the opinion of the Chief Investigator (or nominated representative) is that the event was:
▪ 'related' – that is, resulted from administration of any of the research procedures;
▪ 'unexpected' – that is, the type of event is listed in the protocol as an expected occurrence.

Study sites (Principal investigator) will complete the SAE form and fax/scan it to the Trial manager within one working day of becoming aware of an SAE. The form will then be forwarded to the Chief Investigator for assessment and reports of related and unexpected SAEs will be submitted within 15 days of the Chief Investigator becoming aware of the event to main REC.

When required: the CI can decide to send anonymised details of the event to the TMG for a second opinion to reach consensus of an SAE classification. If the CI, PI or TMG are not in agreement with the "expectedness" classification, both opinions should be recorded on the SAE form.

Systems for SUSAR and SAR reporting should, as far as possible, maintain blinding of individual research staff involved in the running of the trial. Cases that are considered serious, unexpected and possibly, probably or definitely related (i.e. possible SUSARs) would have to be unblinded.

Emotional Support

Some participants may experience distress in answering questions about the impacts of the COVID-19 pandemic. The nature of the questionnaire will be clearly explained on the landing page (participant information sheet) and the consent form. Participants will be able to stop participating in the survey at any time and can skip any questions. Information will be made available at the end of the survey, and after more sensitive questions, for participants who recognise that they are feeling distressed. This will include resources signposting people to support services and helplines:

The NHS website at www.nhs.uk contains resources for supporting your mental health and also has a dedicated helpline for NHS staff affected by COVID-19. To contact the NHS helpline, phone 0300 131 7000, or text FRONTLINE to 85258.
Mind's website at www.mind.org.uk has useful resources to help you cope if you are feeling anxious, worried or isolated.
See Samaritans at www.samaritans.org if you are worried about your mental health. You can also call the Samaritans day or night if you need someone to talk to without judgement on 116 123.
The Every Mind Matters https://www.nhs.uk/oneyou/every-mind-matters/ is a free online resource from the NHS and Public Health England that offers expert help and practical tips on looking after your mental health and wellbeing.
The World Health Organisation at www.who.int has a document detailing mental health and psychosocial considerations during this pandemic.

The research team does not take clinical responsibility for research participants in this study and will not monitor incoming data for risk of self-harm or suicide. This is made clear during the consent process.

There might be some indirect benefits to the participant when taking part. People often value the opportunity to disclose their experiences and feelings. Further, people may feel keen to contribute to research concerning such a stressful and unprecedented situation.

There may be a benefit to their wellbeing and mental state related to the app usage, for those in the intervention arm. Further, all participants will receive a financial incentive related to the degree of participation in this study. Various members of the research team have been running studies into mental health and wellbeing for many years, including many tens of thousands of participants and distress resulting from answering questions like the ones proposed in the current study is extremely rare.

Ethics and Dissemination

22. Research Ethics Approval

Submitted as an ethics amendment to existing project approval.

23. Protocol Amendments

Any protocol amendments will be communicated to all involved parties by email.

24. Consent

Informed online written consent will be sought from all participants via Qualtrics.

25. Confidentiality

All information collected will be managed in the strictest confidence. Participant names will be kept in a separate password protected online database hosted by King's College London, on servers located in the UK, with the file back-up in KCL OneDrive.

26. Declaration of Interests

Study team do not have any conflicts of interests to declare.

27. Access to data

Koa will be provided a clinical study report as aggregated data, as per data sharing agreement.

28. Dissemination Policy

Trial Results: Results will be published in a scientific journal. Aggregated data shared with Koa Health may be used in marketing material thereafter.

Appendices

29. Informed consent materials

References

Broman, J.E., Smedje, H., Mallon, L. and Hetta, J., 2008. The minimal insomnia symptom scale (MISS). *Upsala journal of medical sciences*, *113*(2), pp.131-142.

Goldberg, D.P., Gater, R., Sartorius, N., Ustun, T.B., Piccinelli, M., Gureje, O. and Rutter, C., 1997. The validity of two versions of the GHQ in the WHO study of mental illness in general health care. *Psychological medicine*, *27*(1), pp.191-197.

Koopman, C., Pelletier, K.R., Murray, J.F., Sharda, C.E., Berger, M.L., Turpin, R.S., Hackleman, P., Gibson, P., Holmes, D.M. and Bendel, T., 2002. Stanford presenteeism scale: health status and employee productivity. *Journal of occupational and environmental medicine*, *44*(1), pp.14-20.

Löwe, B., Kroenke, K., Herzog, W. and Gräfe, K., 2004. Measuring depression outcome with a brief self-report instrument: sensitivity to change of the Patient Health Questionnaire (PHQ-9). *Journal of affective disorders*, *81*(1), pp.61-66.

Mundt, J.C., Marks, I.M., Shear, M.K. and Greist, J.M., 2002. The Work and Social Adjustment Scale: a simple measure of impairment in functioning. *The British Journal of Psychiatry*, *180*(5), pp.461-464.

Reilly, M.C., Zbrozek, A.S. and Dukes, E.M., 1993. The validity and reproducibility of a work productivity and activity impairment instrument. *Pharmacoeconomics*, *4*(5), pp.353-365.

Smith, B.W., Dalen, J., Wiggins, K., Tooley, E., Christopher, P. and Bernard, J., 2008. The brief resilience scale: assessing the ability to bounce back. *International journal of behavioral medicine*, *15*(3), pp.194-200.

Spitzer, R.L., Kroenke, K., Williams, J.B. and Löwe, B., 2006. A brief measure for assessing generalized anxiety disorder: the GAD-7. *Archives of internal medicine*, *166*(10), pp.1092-1097.

Tennant, R., Hiller, L., Fishwick, R., Platt, S., Joseph, S., Weich, S., Parkinson, J., Secker, J. and Stewart-Brown, S., 2007. The Warwick-Edinburgh mental well-being scale (WEMWBS): development and UK validation. *Health and Quality of life Outcomes*, *5*(1), pp.1-13.
