# Supplementary material for: Multicentre, England-wide randomised controlled trial of the ‘Foundations’ smartphone application in improving mental health and well-being in a healthcare worker population
Source: Br J Psychiatry. 2023 Feb;222(2):58–66. doi: 10.1192/bjp.2022.103 (PMC10895508; doi:10.1192/bjp.2022.103)
Supplement: Supplementary file 1 [file S0007125022001039sup.zip › S0007125022001039sup003.pdf]

# NHS CHECK - Health & Experiences of staff working at NHS Trusts and Nightingale Hospitals.

Embedded RCT Full Title: Effectiveness of a smartphone app-based intervention in improving stress, resilience, wellbeing and mental health outcomes in a high-risk healthcare worker population during COVID-19: a randomised controlled parallel group trial.

Embedded RCT Short Title: Smartphone app-based mental health and wellbeing intervention for healthcare workers during COVID-19.

Statistical Analysis Plan

Version 1.4: 03/08/2021

Protocol Version 1

ISRCTN18395399

Trial Statistician: Rose Tinch-Taylor

Signature: Date: 03/08/2021

Senior Statistician: Dr. Ben Carter

Signature: Date: 03/08/2021

Chief Investigator: Dr. Sam Gnanapragasam

Signature: Date: 03/08/2021

## Contents

**A) QUANTITATIVE ANALYSIS PLAN .......... 4**
**1. Description of the trial .......... 6**
1.1 Principal research objectives to be addressed .......... 6
Primary objectives .......... 6
Secondary objectives .......... 6
1.2 Trial design including blinding .......... 7
Table 1: Blinding status of personnel .......... 9
Figure 1. Trial design flow diagram .......... 10
1.3 Method of allocation of groups .......... 11
Sequence generation .......... 11
1.4 Duration of the treatment period .......... 11
1.5 Frequency and duration of follow-up .......... 11
1.6 Visit windows .......... 12
1.7 Data collection .......... 12
Eligibility screening .......... 12
Measures .......... 13
1.8 Sample size estimation .......... 14
1.9 Brief description of proposed analyses and any pre-analysis statistical checks required .......... 15
**2. Data analysis plan – Data description .......... 16**
2.1 Recruitment and representativeness of recruited participants .......... 16
2.2 Baseline comparability of randomised groups .......... 18
2.3 List of anticipated intercurrent events .......... 18
2.4 Adherence to allocated treatment and treatment fidelity .......... 18
2.5 Loss to follow-up and other missing data .......... 18
Missing items in scales and subscales .......... 19
Missing baseline data .......... 19
Missing outcome data .......... 19
2.6 Adverse event reporting .......... 19
2.7 Assessment of outcome measures (unblinding) .......... 20
2.8 Descriptive statistics for outcome measures .......... 20
2.9 Listing of concomitant therapies .......... 20
**3. Data analysis plan – Inferential analysis .......... 20**
3.1 Main analysis of treatment differences .......... 20
3.2 Analysis of primary outcomes .......... 21
3.3 Analysis of secondary outcomes .......... 22
3.4 Statistical considerations .......... 22
Time points .......... 22
Stratification .......... 22
Method for handling multiple comparisons .......... 22
Method for handling non-compliance (per protocol/CACE analyses) .......... 22
Model assumption checks .......... 23
3.5 Populations under investigation .......... 23
3.6 Sensitivity analyses .......... 23
Per protocol population .......... 23

Non-ignorable missing outcome data........24
Association of drop-out........24
3.7 Planned subgroup analyses........24
3.8 Exploratory analyses........25
**4. Quality Control, and assurance........25**
**5. Software........26**
**B) SCHEDULE OF ASSESSMENTS AND MEASURES........27**
**6. Reference list........29**
**7. Amendments to version 1.0........30**

## A) QUANTITATIVE ANALYSIS PLAN

**Investigators**
Professor Neil Greenberg
Professor of Defence Mental Health. Department of Psychological Medicine, Institute of Psychiatry Psychology and Neuroscience, King's College London

Dr. Sharon Stevelink
Lecturer in Epidemiology. Department of Psychological Medicine, Institute of Psychiatry Psychology and Neuroscience, King's College London

Dr. Ira Madan
Director. Occupational Health, Guys and St Thomas' NHS Foundation Trust

Dr. Danielle Lamb
Senior Research Fellow. Institute of Epidemiology & Health, University College London

Dr. Hannah Scott
Post-Doctoral Researcher. Department of Psychological Medicine, Institute of Psychiatry Psychology and Neuroscience, King's College London

Emilia Souliou
Research Assistant. Department of Psychological Medicine, Institute of Psychiatry Psychology and Neuroscience, King's College London

Siobhan Hegarty
Research Assistant. Department of Psychological Medicine, Institute of Psychiatry Psychology and Neuroscience, King's College London

**Database Manager**
Danny Weston
Database Manager. Department of Psychological Medicine, Institute of Psychiatry Psychology and Neuroscience, King's College London

**Joint Chief Investigators**
Dr. Sam Gnanapragasam
NIHR Academic Clinical Fellow. Department of Psychological Medicine, Institute of Psychiatry Psychology and Neuroscience, King's College London

Professor Simon Wessely
Regius Professor of Psychiatry. King's College London. Director. NIHR Health Protection Research Unit on Emergency Preparedness and Response

**Trial Project Manager**
Rupa Bhundia

Project Manager of NHS CHECK Study. Department of Psychological Medicine, Institute of Psychiatry Psychology and Neuroscience, King's College London

**Trial Statisticians**

Rose Tinch-Taylor
Statistician. Department of Biostatistics and Health Informatics, Institute of Psychiatry Psychology and Neuroscience, King's College London

Dr Ben Carter
Senior Lecturer in Biostatistics. Department of Biostatistics and Health Informatics, Institute of Psychiatry Psychology and Neuroscience, King's College London

**Trial Researchers**

Dr. Hannah Scott
Post-Doctoral Researcher. Department of Psychological Medicine, Institute of Psychiatry Psychology and Neuroscience, King's College London

Emilia Souliou
Research Assistant. Department of Psychological Medicine, Institute of Psychiatry Psychology and Neuroscience, King's College London

Siobhan Hegarty
Research Assistant. Department of Psychological Medicine, Institute of Psychiatry Psychology and Neuroscience, King's College London

# 1. Description of the trial

The NHS CHECK trial aims to evaluate whether the use of the mobile application Foundations improves stress, wellbeing, anxiety, depression, functioning, resilience, and sleep in a real-world, high-risk healthcare worker cohort.

The randomised control trial will explore psychological and social well-being measures for NHS staff. Participants will include those working within medical, nursing, midwifery, allied health professionals, support, administrative and management roles.

The eight-week trial will be conducted within 16 NHS trusts with catchment areas ranging from high density urban to rural. It will compare participants randomised to the Foundations mobile application trial arm with participants randomised to the control arm (waitlist-for-application).

## *1.1 Principal research objectives to be addressed*

**Primary objective**

To determine the effectiveness of the Foundations mobile application on general (non-psychotic) psychiatric morbidity in a real-world, high-risk healthcare worker cohort, as measured by the total score on the 12-item General Health Questionnaire, Goldberg et al., 1992 [1].

**Secondary objectives**

To determine the effectiveness of the Foundations mobile application in improving stress, wellbeing, anxiety, depression, functioning, resilience, presentism, and sleep.

## 1.2 Trial design including blinding

NHS CHECK is a multi-trust, UK wide, researcher only blinded, parallel-group randomised controlled trial comparing the mental wellbeing mobile application Foundations to the waitlist-for-application control group in a 1:1 ratio.

The randomised control trial will use completed baseline data from the NHS CHECK cohort study [2] to identify individuals working within NHS trusts who meet the eligibility criteria and have consented to be contacted for further research.

Identified individuals will be contacted and invited to the trial. Participants who want to take part will be invited to complete the baseline assessment where eligibility will be reassessed, and consent provided.

All eligible and consented participants will then be randomised to receive the Foundations mobile application or to be part of the wait-list control arm. All participants from both arms will follow treatment-as-usual conditions and will also be asked to complete assessments at week 4 (post-randomisation) and week 8 (post-randomisation) comprising the measures detailed below. All participants will receive the same emails to complete the week 4 and week 8 follow-up assessments; these will be sent three times in week 4 and three times in week 8.

**Interventions under investigation**

Control (Waitlist-for-application)

1. All participants in the waitlist-for-application control arm will continue treatment-as-usual.
2. At the end of the eight-week trial period, following the completion of the measures, waitlist-for-application participants will receive access to the application. They will be contacted by NHS CHECK and sent access links and login details

Intervention (Foundations mental wellbeing mobile application):

1. All participants in the intervention arm will continue treatment-as-usual.
2. All participants will also be invited to use the Foundation application to complete interactive activities and programmes designed to build resilience, manage stress, and improve sleep using the Foundations mobile application. There will be no prescription related to the number or type of activity or program to undertake. The application includes psychoeducational content aimed at promoting well-being based on cognitive behavioural therapy (CBT), mindfulness-based CBT, insomnia targeted CBT, relaxation techniques and positive psychology, with a mixture of standalone activities and programmes. Activities are delivered through a variety of formats including journaling, learning articles and slides, audios, and quizzes; a programme comprises a sequenced set of activities.
3. Participants in the intervention arm will be contacted with scheduled emails up to twice a week from the Foundations App with reminders to encourage use (in line with regular use); participants may also opt in within the application to receive push notifications up to three times per week to encourage application use, one of which will be personalised to those in the study. Based upon adherence/use in week one and two, further engagement may occur such as the study team contacting participants via email to encourage participants to continue using the application.

**Blinding and planned unblinding**

The participants will know of the allocation; it is not possible to blind participants due to the nature of the intervention. The Senior Statistician, Investigators and one of the Chief Investigators will be blind to allocation until the end of the trial when planned unblinding will occur. The Trial Statistician will be fully blind until the first draft of the SAP is approved by the Senior Statistician and Chief Investigator, then will be pseudo blind (able to see participant outcome data coded A/B) following King’s College London Clinical Trials Unit (KCTU) Standard Operating Procedures

(SOPs). Blinding will be maintained by ensuring participant data is stored in separate databases from the randomisation data and held by the four fully unblinded researchers.

**Table 1: Blinding status of personnel**

| **Role** | **Blinding status throughout trial** | **Reasoning and timing of planned unblinding** |
|---|---|---|
| **Chief Investigator (Professor Simon Wessely)** | Fully blinded | On conclusion of the trial results to the Trial Management Group. |
| **Chief Investigator (Dr. Sam Gnanapragasam)** | Unblinded | Due to assisting with queries regarding the randomisation of participants. |
| **Co-investigators (Professor Neil Greenberg; Dr. Sharon Stevelink; Dr. Ira Madan; Dr. Danielle Lamb)** (excluding HS, SH and ES who are also Trial Researchers and listed below) | Fully blinded | On conclusion of the trial results to the Trial Management Group. |
| **Senior Statistician (Dr Ben Carter)** | Fully blinded | On conclusion of the trial results to the Trial Management Group. |
| **Trial Statistician (Rose Tinch-Taylor)** | Fully blinded / partially blinded | On approval of the SAP to the Trial Management Group. |
| **Trial Project Manager (Rupa Bhundia)** | Unblinded | Due to randomising participants and management of the randomisation database. |
| **Trial Researchers (Dr. Hannah Scott; Siobhan Hegarty; Emilia Souliou)** | Unblinded | Due to randomising participants and management of the randomisation database. |

## *Figure 1. Trial design flow diagram*

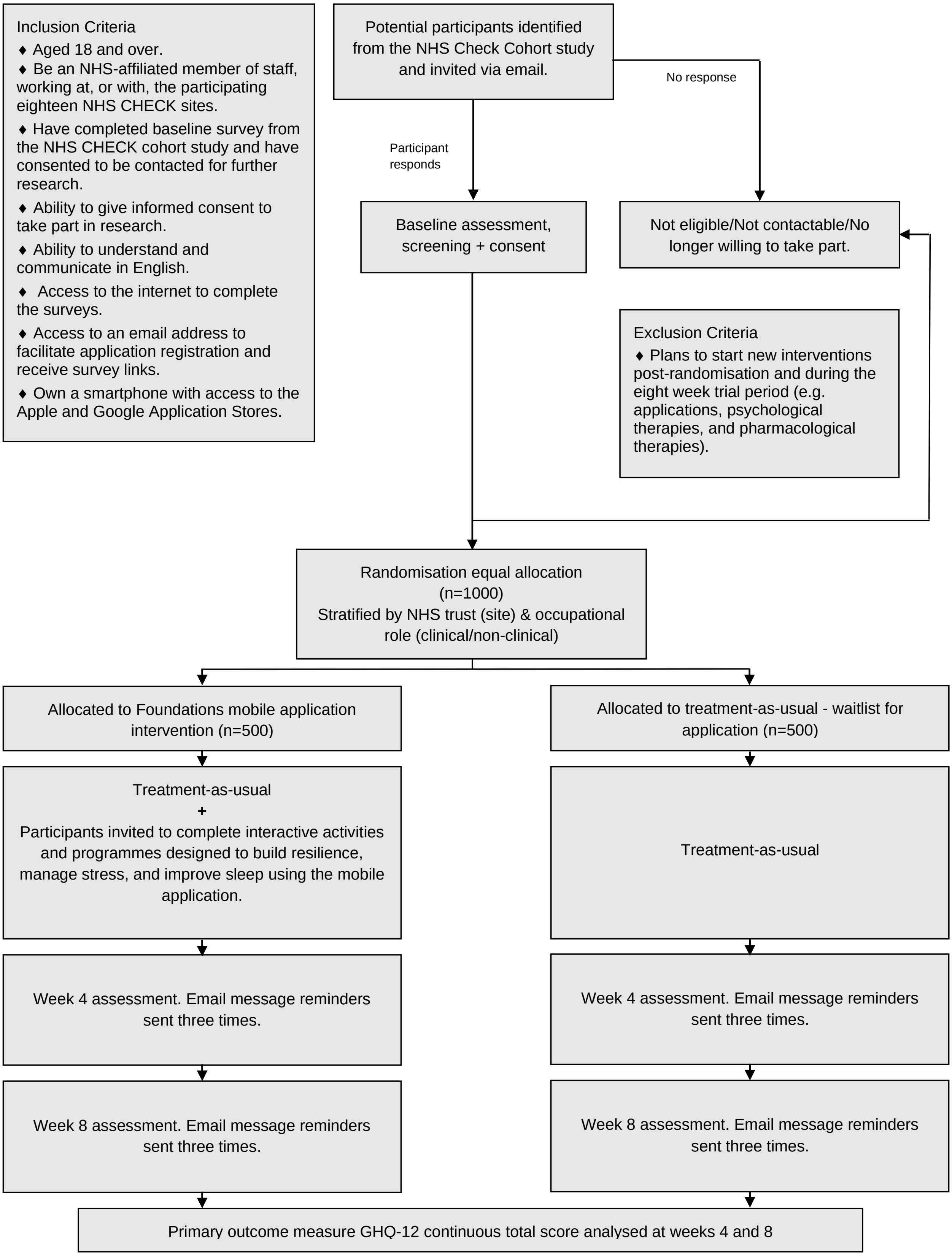

## *1.3 Method of allocation of groups*

**Sequence generation**

Following completion of the baseline questionnaire, participants will be randomised to one of the two treatment arms. Randomisation will be done in a 1:1 ratio. Randomisation is at the participant level and is performed using an independent online system based at the King's Clinical Trials Unit (King's CTU) at King's College London.

Randomisation is stratified by NHS trust (site) and occupational role (clinical or non-clinical), using a varying permuted block design (2 and 4 units) to ensure that equal numbers of participants are allocated to the two arms.

**Process of allocation**

The procedure is as follows:

On receipt of the baseline questionnaire, three fully unblinded Trial Researchers electronically submit details of each participant to the CTU. This includes participant ID, NHS trust (site), occupational role, first initial and date of birth. The system immediately notifies the relevant researcher and records the randomisation outcome. The Trial Project Manager does not receive the randomisation outcome.

The sequence will be concealed from the investigators including the Chief Investigators and Statisticians.

## *1.4 Duration of the treatment period*

The treatment period will be for 8 weeks from 0 weeks post-randomisation to 8 weeks post-randomisation.

## *1.5 Frequency and duration of follow-up*

Participants will complete follow up measures at week 4 post-randomisation and at week 8 post-randomisation.

## 1.6 Visit windows

The following visit windows apply to the assessments carried out in the trial:

i. Pre-randomisation (baseline) assessment; 1 to 0 weeks pre-randomisation.

ii. Post-randomisation week 4 outcome assessment; 4 weeks post randomisation ± 9-day window, i.e. 19 and 37 days post-randomisation.

iii. Post-randomisation week 8 outcome assessment; 8 weeks post-randomisation ± 9-day window, i.e. 47 and 65 days post-randomisation.

A window of ± 9 days for early or late measurements is considered acceptable. If recorded outcome k is late, recorded outcome k+1 will be measured from visit k.

## 1.7 Data collection

**Eligibility screening**

Inclusion:

1. Aged 18 and over.
2. Be an NHS-affiliated member of staff, working at, or with, the participating 16 NHS CHECK sites.
3. Completed the baseline assessment for the NHS CHECK Cohort study and have consented to be contacted for further research.
4. Ability to provide informed consent to take part in research.
5. Ability to understand and communicate in English.
6. Access to the internet to complete the assessment.
7. Access to an email address to facilitate application registration and receive assessment links.
8. Own a smartphone with access to the Apple or Google application stores.

Exclusion:

1. Plans to start new interventions post-randomisation and during the eight-week trial period (i.e. wellbeing applications, psychological therapies, and pharmacological therapies).

**Measures**

A timeline of data collected is given in B) SCHEDULE OF ASSESSMENTS AND MEASURES. What follows is a brief overview to aid understanding of the analysis plan.

**Primary outcome measure**

1. The GHQ-12 is a 12-item scale which screens for general (non-psychotic) psychiatric morbidity. The GHQ-12 will be scored 0 1 2 3 and summed, resulting in a 0-36 score range with a higher score indicating worse mental health, Goldberg et al., 1992 [1].
   Missing data will be dealt with as per Section 2.5 below.

**Secondary outcome measures**

2. The Brief Resilience Scale (BRS) continuous total score to measure resilience is a 6-item participant self-report measure, which assesses an individual's ability to bounce back or recover from stress, Smith et al., 2008 [3].
3. The Generalized Anxiety Disorder (GAD-7) continuous total score is a well validated and widely used 7-item measure of anxiety. A change of 4 or more on the GAD has been found to be clinically significant across anxiety disorders, Spitzer et al., 2006 [4].
4. The Patient Health Questionnaire (PHQ-9) continuous total score is the 9-item depression module from the full PHQ. A score of 10 to 27 indicates moderate to severe depression. There is strong evidence for the validity of the PHQ-9 as a brief measure of depression severity, Kroenke et al., 2001 [5].
5. The Short Warwick-Edinburgh Mental Well-being Scale (SWEMWBS) continuous (converted) total score is a 7-item measure to enable the monitoring of subjective well-being and psychological functioning compared to the general population (all items are worded positively and address aspects of positive mental health), Tennant et al., 2007 [6].
6. The 5-item Work and Social Adjustment Scale (WSAS) continuous total score. A WSAS score above 20 appears to suggest moderately severe or worse psychopathology. Scores between 10 and 20 are associated with significant

functional impairment but less severe clinical symptomatology, Mundt et al., 2002 [7].

7. The 6-item Stanford Presenteeism Scale (SPS-6) continuous total score, Koopman et al., 2002 [8].
8. The Minimal Insomnia Symptom Scale (MISS) continuous total score is a 3-item scale which provides a brief measure of sleeping difficulties, Broman et al., 2008 [9].

**Alongside the outcome measures listed above, data will be collected for:**

9. Eligibility and consent.
10. Demographic data to include sex, occupation, ethnicity, and country of birth.
11. Current Psychological & Pharmacological Support (CPPS), including use of psychotropic medication, psychological therapies, and mobile wellbeing applications.
12. Moral Injury Event Scale (MIES).
13. COVID-19 stressors that have significantly impacted participants in the six months prior to baseline data collection, and in the previous month for the 4 and 8-week assessments.
14. Reported adverse events.
15. Adherence.

## *1.8 Sample size estimation*

For this parallel arm, 1:1 ratio design, we originally aimed to enrol 700 participants to give 80% power at the 5% level of significance, assuming a standardized effect size of 0.3 and attrition of 50% following meta-analyses evidence for smartphone-delivered interventions studies, Linardon & Fuller-Tyszkiewicz, 2020 [10].

Following initial recruitment, the proportion of individuals within the treatment arm who failed to have fidelity (by not downloading the mobile application) was higher than anticipated. This emerging evidence has thus informed our decision to re-evaluate the sample size and increase recruitment to 1000 participants. We have

assumed a standardized effect size of 0.3 for 5% level of significance and 80% power. We have also assumed that for participants who complete the 8-week follow-up, 60% of them will have had fidelity to the treatment.

## *1.9 Brief description of proposed analyses and any pre-analysis statistical checks required*

Analyses will be carried out by the Trial Statistician.

The primary analyses will use the modified intention-to-treat population. A mixed effects multivariable regression model will be used, conditioning on baseline disease severity and key confounders with a random intercept fitted for both NHS trust (site) and participant. Linear multivariable analyses will be presented as an adjusted mean difference between the Foundations application versus control with the associated 95% confidence interval and p-value. Logistic multivariable analyses will be presented as adjusted odds ratios (OR) on the raw scale with 95% confidence intervals and p-value.

# 2. Data analysis plan – Data description

## *2.1 Recruitment and representativeness of recruited participants*

A CONSORT flow chart will be constructed [11] – see Figure 2. This will include the number of eligible participants, the number of participants agreeing to enter the trial, the number of participants refusing, then by treatment arm: the number of participants who received the allocated intervention, the number of participants who downloaded the application, the number of participants who adhered to the minimum usage in week 1 and week 2, the number continuing through the trial, the number withdrawing, the number lost to follow-up and the numbers excluded/analysed.

**Figure 2. Template CONSORT diagram for NHS CHECK trial**

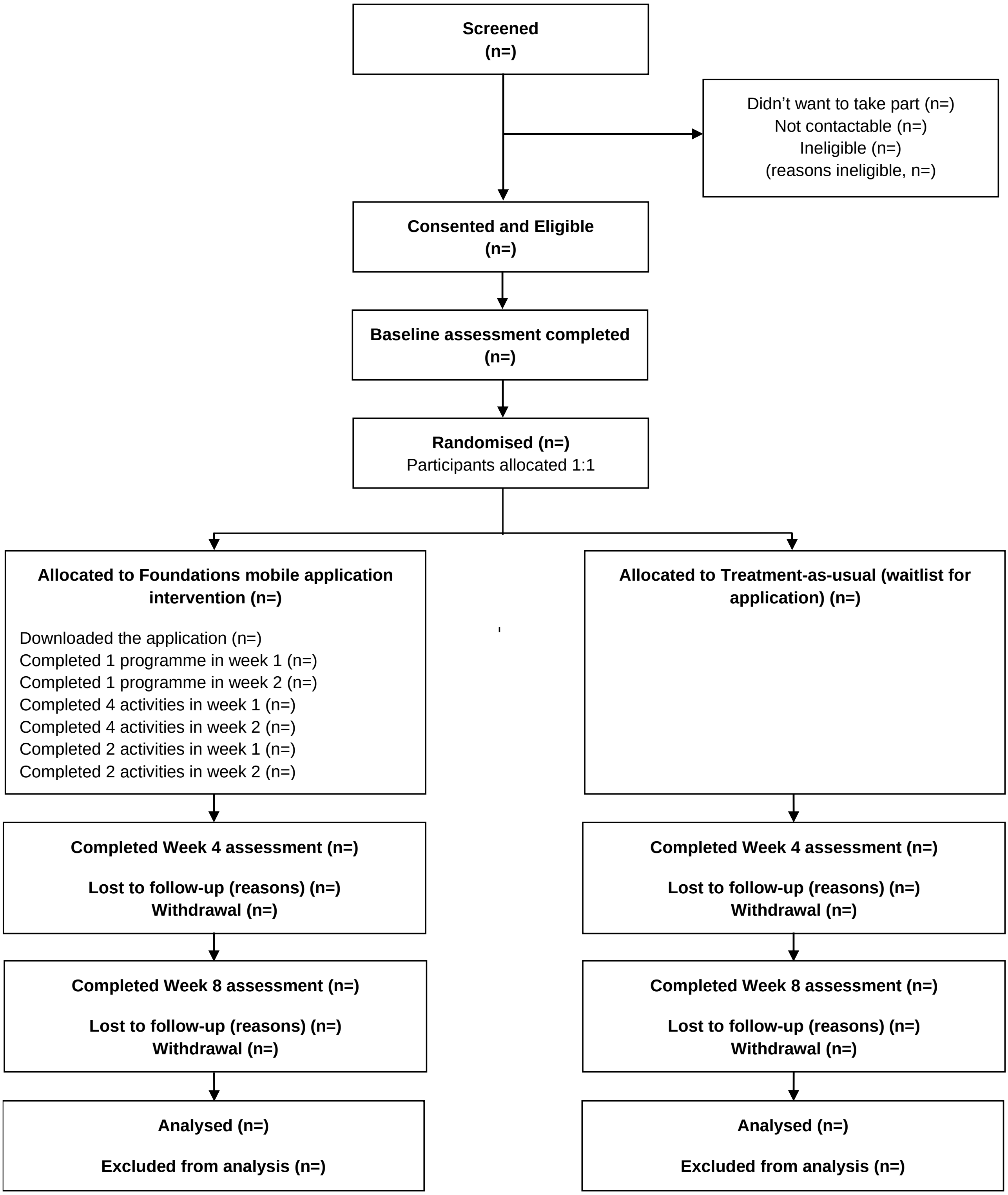

## 2.2 Baseline comparability of randomised groups

All outcome measures listed in section 1.7 above will be summarised overall and by trial arm: means and standard deviation or numbers and proportions as appropriate. All other data collected at baseline will be summarised in the same way. No significance testing will be used to test baseline differences between the randomised treatment groups, Rombach et al [12].

## 2.3 List of anticipated intercurrent events

Intercurrent events (ICEs) that occur after randomisation and affect the occurrence or interpretation of outcome measures after the event include:

• Trial discontinuation

## 2.4 Adherence to allocated treatment and treatment fidelity

Adherence to treatment will be described by the number and proportion of participants that successfully downloaded the mobile application.

As this will unblind the Trial Statistician, coding of this variable will be conducted at the end of the analysis.

Further descriptions will be provided for the treatment arm: means and standard deviation or numbers and proportions as appropriate for:

1. Number of participants who have downloaded the mobile application.
2. Number of participants who completed 4 activities or 1 programme in week 1 and in week 2.
3. Number and type of programmes and activities selected and completed.
4. Number of participants who have completed 29 distinct activities.
5. Average amount of time spent using the application overall and weekly.

## 2.5 Loss to follow-up and other missing data

The proportions of participants with variable values missing or unused will be summarised in each arm and at each time point.

The baseline characteristics of those missing follow up will be compared to those with completed follow up.

**Missing items in scales and subscales**

The number (%) with complete data will be reported. Where present, missing value guidance provided for scales will be used. Where this is not possible, scales will be pro-rated for an individual if 25% or fewer items are missing. For example, in a scale with 10 items, pro-rating will be applied to individuals with 1 or 2 items missing. The average value for the 8 or 9 complete items will be calculated for that individual and used to replace the missing values. The scale score will be calculated based on the complete values and these replacements, S. F. Assmann et al, 2000 [13].

**Missing baseline data**

We anticipate negligible missing baseline covariate data. The number of participants with complete data will be reported and any missing baseline covariate data may be imputed using a method suitable to the variable as per the recommendations of White and Thompson, 2005 [14].

**Missing outcome data**

Analyses will be undertaken assuming outcomes are missing-at-random (MAR) and using all available data. This allows drop-out to be related to treatment group, stratification factors, and baseline severity.

Sensitivity analyses will be carried out to assess the association of drop-out with occupational role (clinical or non-clinical) and demographics and their inclusion as additional covariates.

## *2.6 Adverse event reporting*

Serious adverse events (SAE) will be summarised by treatment arm.

### 2.7 Assessment of outcome measures (unblinding)

Researchers and the Senior Statistician are being kept blind to treatment allocation.

### 2.8 Descriptive statistics for outcome measures

Each of the outcome measures will be described by trial arm at week 4 and week 8. Means and standard deviations or medians and interquartile ranges will be used for the continuous variables; Q-Q plots will be used to assess whether the distribution of a variable is normal.

### 2.9 Listing of concomitant therapies

A departure from the trial's regimen would include participants who start new interventions post-randomisation and during the eight-week trial period (mobile applications, psychological therapies, and pharmacological therapies). All concomitant therapies will be listed by treatment arm.

## 3. Data analysis plan – Inferential analysis

### 3.1 Main analysis of treatment differences

The main analysis will use a modified intention-to-treat (ITT) population unless otherwise specified (i.e. all randomised participants who have reported to at least one of the post randomised time-points will be included according to their allocated trial arm, irrespective of treatment received).

The analyses will use a mixed effects multivariable regression model, conditioning on baseline severity and covariates. A random intercept will be fitted for both participant and site to explain heterogeneity across these effects.

Linear multivariable analyses will be presented as mean differences (or adjusted mean differences) between the Foundations application versus control with the associated 95% confidence interval and p-value. Logistic multivariable analyses will

be presented as odds ratios (OR) on the raw scale with associated confidence interval and p-value.

Analysis will be carried out using Stata version 16 [15]. The significance level of 5% (two-sided) will be used for all outcomes.

The Senior Statistician will remain blind until the main analyses are complete. Any analyses that cannot be performed blind will be done at the end of the final analysis to preserve blinding for as long as possible.

## *3.2 Analysis of primary outcomes*

The analysis population will include all randomised participants who have completed one of the post-randomisation time points.

The primary outcome is the 12-item General Health Questionnaire (GHQ-12) score at 4- and 8-weeks post-randomisation. The GHQ-12 will be scored 0 1 2 3 and summed, resulting in a 0-36 score range with a higher score indicating worse mental health.

A mixed effects multivariable regression model will be used to estimate the difference in mean GHQ-12 score between arms, with the temporal effect of the 4-week and 8-week assessments as independent variables and a random intercept at the participant level and at the site level. The model will also include treatment group, baseline GHQ-12 score, age, sex, ethnicity, occupational role (clinical or non-clinical), current use of other mental health application (yes or no), current use of mental health/wellbeing medication (yes or no), and current use of psychological or talking therapy (yes or no) as covariates.

Modelling assumptions will be checked. See 3.1.3 below, under model assumption checks.

Sensitivity analyses will be used to assess the robustness of conclusions to non-ignorable missing outcome data, [16].

## *3.3 Analysis of secondary outcomes*

As all secondary outcomes will be measured at both the 4-week and 8-week time-points, treatment effects for all secondary outcomes will lend themselves to the same analysis as described above for the primary outcome. These secondary outcomes will be assessed using similar modelling techniques, employing generalisations to non-normal data where necessary or transformation of the outcome variable.

## *3.4 Statistical considerations*

**Time points**

Each outcome measure for participants will be recorded at three time points: pre-randomisation (baseline) and 4- and 8-weeks' post randomisation.

**Stratification**

Randomisation is stratified by NHS trust (site) and occupational role (clinical or non-clinical). Therefore, these variables will be included as factors in the modelling process as detailed in 3.1.1 above.

**Method for handling multiple comparisons**

No formal adjustment of p-values for multiple testing or as a consequence of multiple comparisons will be made. However, care will be given to the interpretation of inference for the numerous secondary outcomes.

**Method for handling non-compliance (per protocol/CACE analyses)**

In addition to the primary modified Intention-to-Treat (mITT) analysis, the effect of using the mobile application as defined in 2.4 above will be estimated.

If non-compliance with the active treatment is high, a complier average causal effect (CACE) analysis will be considered.

**Model assumption checks**
The models assume normally distributed outcomes; this will have been checked when describing the data and if substantial departures from normality occur, transformations will be considered. Residuals will be plotted to check for normality and inspected for outliers.

## *3.5 Populations under investigation*

**Primary outcome population – Modified Intention-to-Treat (mITT)**
The primary analyses will use the modified intention-to-treat (mITT) population i.e. all randomised participants who report to at least one of the post-randomisation time-points, irrespective of treatment received.

**Per-protocol Population (PPP)**
The per protocol population (PPP) will exclude patients defined as protocol violators (i.e. those that fail to have fidelity by not downloading the mobile application), or reporting outcome data outside of the specified visit windows.

## *3.6 Sensitivity analyses*

**Per protocol population (PPP)**
The PPP will be assessed and excludes participants defined as protocol violators (those that fail fidelity). Sensitivity analyses on the PPP will analyse the primary outcome using the same analysis described in 3.1.1 above, and will:

1. Exclude participants who have not downloaded the mobile application or reported outcome data outside of the visit windows.
2. Exclude participants who have not completed at least one programme in week 1 and at least one programme in week 2, post-randomisation. Participants will also be excluded if they reported outcome data outside of the visit windows.

3. Exclude participants who have not completed a minimum of four activities in week 1 and a minimum of four activities in week 2, post-randomisation. Participants will also be excluded if they reported outcome data outside of the visit windows.
4. Exclude participants who have not completed a minimum of two activities in week 1 and a minimum of two activities in week 2, post-randomisation. Participants will also be excluded if they reported outcome data outside of the visit windows.

**Non-ignorable missing outcome data**

Sensitivity analyses will be used to assess the robustness of conclusions to non-ignorable missing outcome data using the ***rctmiss*** package in Stata 16 [15]. The primary analysis will be carried out under a range of assumptions about the missing data, where the data and missingness are modelled jointly using a pattern-mixture model [16].

**Association of drop-out**

Sensitivity analyses will be carried out on the primary analysis to assess the association of drop-out with occupational role (clinical or non-clinical) and baseline demographic variables and their inclusion as additional covariates.

## *3.7 Planned subgroup analyses*

Given that this study is not powered to detect statistically significant between-group differences, subgroup analyses are not required. However, if recruited sample size allows, we will explore the following comparisons:

1. Gender differences (male vs female)
2. Occupational role (clinical, non-clinical)
3. Age (18-29, 30-39, 40-49, 50-59, 60+)
4. Baseline GHQ-12 score (cut-off at 12)
5. Consultation with GP or Psychiatrist for mental health or stress-related difficulties in previous 6 months (yes or no)

6. Any self-described mental health diagnosis (yes or no)
7. Self-described mental health diagnosis of depression (yes or no)
8. Self-described mental health diagnosis of anxiety disorder (yes or no)
9. Moral injury as taken from the Moral Injury Event Scale (overall binary variable if any item is yes)
10. Current use of mental health/wellbeing mobile application (yes or no)
11. Current use of physical health mobile application (yes or no)
12. Current use of mental or physical health mobile application (yes or no)
13. Current use of mental health/wellbeing medication (yes or no)
14. Current use of psychological or talking therapy (yes or no)
15. Current use of any treatment (mental health mobile application, medication or psychological support) (yes or no)

This analysis will be clearly stated as exploratory in the primary paper/report and will be interpreted accordingly.

### *3.8 Exploratory analyses*

Exploratory analyses will be conducted on the following Covid-19 stressors. These will be summarised overall and by trial arm: means and standard deviation or numbers and proportions as appropriate.

1. Loss of family income.
2. Problems managing your finances.
3. Own illness due to COVID-19.
4. Illness of a family member or friend due to COVID-19.
5. Bereavement due to COVID-19.
6. Caring role for a child/children.
7. Caring role for a dependent.

## 4. Quality Control, and assurance

This study will be reported following the KCTU Standard Operating Procedures (SOP). These processes include developing the protocol and blinding (ST-06), reporting (ST-07), data manipulation (ST-08), statistical document retention (ST-03).

The primary analysis as outlined in this SAP will be checked by the Senior Statistician prior to the statistical report being sent to the Chief Investigators as per KCTU SOP ST-04 (Statistical QA) v1.0. This will be done by review of the syntax code, output files and final report. A meeting will be held with the Trial Statistician to discuss these and how to implement any changes if required. A sign off sheet will then be completed.

## 5. Software

Randomisation system: Randomisation is performed using an independent online system based at the King's Clinical Trials Unit (KCTU) at King's College London.

Outcome data: Online assessment responses will be collected using Qualtrics survey software which will be hosted by King's College London, on servers located in the UK. Data required for analysis will be downloaded in pseudonymised form and stored on KCL OneDrive. Any interactions with Qualtrics will be logged and audited by a designated researcher. The valid Data Protection and Security Toolkit from the King's Centre for Military Health Research at King's College London will be followed during rollout.

Statistical analysis: Stata 16 [15] will be used for data manipulation, description analysis and inferential analyses. R [17] may additionally be used for production of graphs, tables, and reports.

## B) SCHEDULE OF ASSESSMENTS AND MEASURES

| # | Form | Screening assessment | Pre-randomisation assessment | Post-randomisation assessment (4 week) | Post-randomisation assessment (8 week) | Type | Administered to | Administration time |
|---|---|---|---|---|---|---|---|---|
| Qualtrics database | | | | | | | | |
| 1 | NHS CHECK cohort baseline assessment (demographic data and Moral Injury Event Scale (MIES)) | X | | | | Online questionnaire | Participant self-report | 5-10 mins |
| 2 | General Health Questionnaire (GHQ-12) | | X | X | X | Online/in-app rating scale | Participant self-report | 2 mins |
| 3 | Brief Resilience Scale (BRS) | | X | X | X | Online/in-app rating scale | Participant self-report | 1-2 mins |
| 4 | Generalized Anxiety Disorder (GAD-7) | | X | X | X | Online/in-app rating scale | Participant self-report | 1-2 mins |
| 5 | Patient Health Questionnaire (PHQ-9) | | X | X | X | Online/in-app rating scale | Participant self-report | 1-2 mins |

| | | | | | | | | |
|---|---|---|---|---|---|---|---|---|
| 6 | Short Warwick Edinburgh Mental Well-being Scale-7 (SWEMWBS) | | X | X | X | Online/in-app rating scale | Participant self-report | 1-2 mins |
| 7 | Work and Social Adjustment Scale | | X | X | X | Online/in-app rating scale | Participant self-report | 2 mins |
| 8 | Stanford Presenteeism Scale (SPS-6) | | X | X | X | Online/in-app rating scale | Participant self-report | 1-2 mins |
| 9 | Minimal Insomnia Symptom Scale (MISS) | | X | X | X | Online/in-app rating scale | Participant self-report | 1 min |
| 10 | Current Psychological & Pharmacological Support (CPPS) | | X | X | X | Online/in-app questionnaire | Participant self-report | 1-2 mins |
| 11 | COVID-19 stressors | | X | X | X | Online/in-app questionnaire | Participant self-report | 1-2 mins |
| KCL Microsoft OneDrive | | | | | | | | |
| 12 | Eligibility and consent | | X | | | Online/in-app questionnaire | Participant | 2 mins |
| 13 | Adverse events | | When required | | | n/a | Participant | n/a |
| 14 | Adherence | | | | X | Examination of usage data | Researchers | n/a |

## 6. Reference list

**[1]** Goldberg, D.P., Gater, R., Sartorius, N., Ustun, T.B., Piccinelli, M., Gureje, O. and Rutter, C., 1997. The validity of two versions of the GHQ in the WHO study of mental illness in general health care. Psychological medicine, 27(1), pp.191-197.

**[2]** Danielle Lamb, S. G. (2021). The psychosocial impact of the COVID-19 pandemic on 4,378 UK healthcare workers and ancillary staff: initial baseline data from a cohort study collected during the first wave of the pandemic. London: medrxiv.org.

**[3]** Smith, B.W., Dalen, J., Wiggins, K., Tooley, E., Christopher, P. and Bernard, J., 2008. The brief resilience scale: assessing the ability to bounce back. International journal of behavioral medicine, 15(3), pp.194-200.

**[4]** Spitzer, R.L., Kroenke, K., Williams, J.B. and Löwe, B., 2006. A brief measure for assessing generalized anxiety disorder: the GAD-7. Archives of internal medicine, 166(10), pp.1092-1097.

**[5]** Löwe, B., Kroenke, K., Herzog, W. and Gräfe, K., 2004. Measuring depression outcome with a brief self-report instrument: sensitivity to change of the Patient Health Questionnaire (PHQ-9). Journal of affective disorders, 81(1), pp.61-66.

**[6]** Tennant, R., Hiller, L., Fishwick, R., Platt, S., Joseph, S., Weich, S., Parkinson, J., Secker, J. and Stewart-Brown, S., 2007. The Warwick-Edinburgh mental well-being scale (WEMWBS): development and UK validation. Health and Quality of life Outcomes, 5(1), pp.1-13.

**[7]** Mundt, J.C., Marks, I.M., Shear, M.K. and Greist, J.M., 2002. The Work and Social Adjustment Scale: a simple measure of impairment in functioning. The British Journal of Psychiatry, 180(5), pp.461-464.

**[8]** Koopman, C., Pelletier, K.R., Murray, J.F., Sharda, C.E., Berger, M.L., Turpin, R.S., Hackleman, P., Gibson, P., Holmes, D.M. and Bendel, T., 2002. Stanford presenteeism scale: and employee productivity. Journal of occupational and environmental medicine, 44(1), pp.14-20.

**[9]** Broman, J.E., Smedje, H., Mallon, L. and Hetta, J., 2008. The minimal insomnia symptom scale (MISS). Upsala journal of medical sciences, 113(2), pp.131-142.

**[10]** Linardon & Fuller-Tyszkiewicz. Attrition and adherence in smartphone-delivered interventions for mental health problems: A systematic and meta-analytic review. J Consult Clin Psychol 2020 Jan; 88(1):1-13.

**[11]** Moher D, Schulz KF, Altman DG. The CONSORT statement: revised recommendations for improving the quality of reports of parallel-group randomised trials. Lancet 2001 Apr 14;357(9263):1191-4.

**[12]** Multiple imputation for patient reported outcome measures in randomised controlled trials: advantages and disadvantages of imputing at the item, subscale or composite score level Ines Rombach1,2* , Alastair M. Gray1 , Crispin Jenkinson3 , David W. Murray2 and Oliver Rivero-Arias4

**[13]** S. F. Assmann, S. J. Pocock, L. E. Enos, and L. E. Kasten. Subgroup analysis and other (mis)uses of baseline data. Lancet. 2000 Mar 25;355(9209)

**[14]** White IR, Thompson SG. Adjusting for partially missing baseline measurements in randomized trials. Stat Med 2005 Apr 15;24(7):993-1007.

**[15]** StataCorp, Stata Statistical Software: Release 15. College Station, TX: StataCorp LLC, 2017.

**[16]** R Development Core Team, R: A language and environment for statistical computing. R Foundation for Statistical Computing, Vienna, Austria, 2008.

**[17]** Nash WP, Marino Carper TL, Mills MA, Au T, Goldsmith A, Litz BT. Psychometric Evaluation of the Moral Injury Events Scale. Mil Med. 2013 Jun 1;178(6):646–52.

## 7. Amendments to version 1.0

**Version 1.1:**
Sensitivity analysis amended to define and extend PPP definitions. Descriptions for adherence to allocated treatment further clarified together with the subgroup analyses. Primary outcome measure GHQ-12 amended to use Likert scoring. One of the chief-investigator's blinding status amended. Minor changes to wording to bring in line with NHS CHECK protocol.

**Version 1.2:**
Expanded descriptions of measures. Updated threshold for Moral Injury Event Scale.

**Version 1.3:**
Redefined age categories. Updated Moral Injury Event Scale to be classified using a binary variable, as per the NHS cohort study.

**Version 1.4:**
Expanded explanation of statistical models.
